# Inhibition of tartrate-resistant acid phosphatase 5 can prevent cardiac fibrosis after myocardial infarction

**DOI:** 10.1186/s10020-024-00856-1

**Published:** 2024-06-15

**Authors:** Shujun Yang, Liying Pei, Zijie Huang, Yinsheng Zhong, Jun Li, Yinghui Hong, Huibao Long, Xuxiang Chen, Changqing Zhou, Guanghui Zheng, Chaotao Zeng, Haidong Wu, Tong Wang

**Affiliations:** 1 Department of Emergency, the Eighth Affiliated Hospital of Sun Yat-sen University, Shenzhen, 518003 Guangdong P. R. China; 2 Department of Emergency, Sun Yat-sen Memorial Hospital of Sun Yat-sen University, Guangzhou, 510120 Guangdong P. R. China

**Keywords:** Myocardial infarction, Cardiac fibrosis, Tartrate-resistant acid phosphatase 5 (ACP5), Cardiac fibroblasts (CFs), Glycogen synthase kinase-3β (GSK3β)/β-catenin signaling pathway

## Abstract

**Background:**

Myocardial infarction (MI) leads to enhanced activity of cardiac fibroblasts (CFs) and abnormal deposition of extracellular matrix proteins, resulting in cardiac fibrosis. Tartrate-resistant acid phosphatase 5 (ACP5) has been shown to promote cell proliferation and phenotypic transition. However, it remains unclear whether ACP5 is involved in the development of cardiac fibrosis after MI. The present study aimed to investigate the role of ACP5 in post-MI fibrosis and its potential underlying mechanisms.

**Methods:**

Clinical blood samples were collected to detect ACP5 concentration. Myocardial fibrosis was induced by ligation of the left anterior descending coronary artery. The ACP5 inhibitor, AubipyOMe, was administered by intraperitoneal injection. Cardiac function and morphological changes were observed on Day 28 after injury. Cardiac CFs from neonatal mice were extracted to elucidate the underlying mechanism *in vitro.* The expression of ACP5 was silenced by small interfering RNA (siRNA) and overexpressed by adeno-associated viruses to evaluate its effect on CF activation.

**Results:**

The expression of ACP5 was increased in patients with MI, mice with MI, and mice with Ang II-induced fibrosis in vitro. AubipyOMe inhibited cardiac fibrosis and improved cardiac function in mice after MI. ACP5 inhibition reduced cell proliferation, migration, and phenotypic changes in CFs in vitro, while adenovirus-mediated ACP5 overexpression had the opposite effect. Mechanistically, the classical profibrotic pathway of glycogen synthase kinase-3β (GSK3β)/β-catenin was changed with ACP5 modulation, which indicated that ACP5 had a positive regulatory effect. Furthermore, the inhibitory effect of ACP5 deficiency on the GSK3β/β-catenin pathway was counteracted by an ERK activator, which indicated that ACP5 regulated GSK3β activity through ERK-mediated phosphorylation, thereby affecting β-catenin degradation.

**Conclusion:**

ACP5 may influence the proliferation, migration, and phenotypic transition of CFs, leading to the development of myocardial fibrosis after MI through modulating the ERK/GSK3β/β-catenin signaling pathway.

**Supplementary Information:**

The online version contains supplementary material available at 10.1186/s10020-024-00856-1.

## Introduction

Myocardial infarction (MI) is the leading cause of cardiovascular disease mortality (Zhang et al. 2023). Although reperfusion strategies have reduced the mortality rate in patients with MI, the long-term mortality rate due to heart failure remains high in MI patients (Eapen et al. 2012). After MI, reparative CFs are activated to form fibrotic scars that maintain the structural integrity of the ventricle. However, excessive interstitial fibrosis leads to impaired cardiac diastolic and systolic function, as well as the occurrence of heart failure (Frangogiannis 2021). Therefore, gaining insight into the intrinsic mechanisms that regulate cardiac fibrosis has the potential to uncover novel therapeutic targets for interventions following MI.

Under normal circumstances, myocardial fibroblasts remain quiescent. After MI, CFs proliferate, migrate to the injured area, and differentiate into myofibroblasts in response to hormones, growth factors, and cytokines (Talman and Ruskoaho 2016). Myofibroblasts synthesize and secrete molecules that promote their migration and phenotypic transition towards a contractile state (Frangogiannis 2019). In addition, myofibroblasts produce collagen, fibronectin, and nonstructural extracellular matrix components to replace necrotic tissue and form fibrotic scars (Prabhu and Frangogiannis 2016; Fan and Kassiri 2021; Frantz et al. 2022). This reparative fibrosis helps maintain the structural integrity of the ventricular wall after MI. However, the process of fibrotic remodeling increases ventricular stiffness, reduces ventricular compliance, and impairs both the systolic and diastolic functions of the heart (Fu et al. 2018; Frangogiannis 2019).

In recent years, studies have shown that the Wnt signaling pathway plays an integral role in the fibrosis in multiple organs(Seo et al. 2019; Yiu et al. 2021; Wang et al. 2024). Glycogen synthase kinase-3β (GSK3β) is a key regulatory factor in the Wnt/β-catenin signaling pathway that maintains the balance between cytoplasmic degradation and nuclear translocation of β-catenin (Guo et al. 2017). Loss of GSK3β leads to the accumulation and nuclear translocation of β-catenin, resulting in the activation of β-catenin-dependent gene programs (Maruyama and Imanaka-Yoshida 2022). It has been reported that inhibiting the expression of the key proteins, namely, GSK3β and β-catenin in the Wnt/ β-catenin signaling pathway can delay cardiac hypertrophy and fibrosis, as well as improve cardiac function (Xiang et al., 2017; Kim et al. 2022).

Tartrate-resistant acid phosphatase 5 (TRAP/ACP5), located at the p13.2-p13.3 site of human chromosome 19, contains a dinuclear iron center (Lausch et al. 2011). ACP5 is a widely distributed, structurally conserved, and multifunctional protein (Hayman 2008). Studies have shown that ACP5 promotes cell proliferation (Bian et al. 2019), migration (Reithmeier et al. 2017), and cellular phenotypic transition (Hu et al. 2020), which are crucial characteristics of fibrotic scar formation. Furthermore, Hu et al. demonstrated that ACP5 promotes pulmonary fibrosis by modulating β-catenin signaling (Hu et al. 2022). However, it remains unclear whether ACP5 plays a role in post-MI fibrosis.

In the present study, in vivo and in vitro experiments were conducted to explore the role of ACP5 in cardiac fibrosis after myocardial infarction. In vivo experiments showed that the ACP5 inhibitor, AubipyOMe, improves cardiac fibrosis after MI. In vitro experiments revealed that ACP5 affects the proliferation, migration, and fibrosis of CFs by influencing the ERK/GSK3β/β-catenin pathway. In summary, the present data support the involvement of ACP5 in the progression of myocardial fibrosis after MI. Therefore, strategies aimed at inhibiting ACP5, such as AubipyOMe, may serve as viable treatments for cardiac fibrosis after MI and have potential in clinical settings.

## Materials and methods

### Clinical blood samples

The inclusion criteria for patients were as follows: over 18 years of age and diagnosed with acute myocardial infarction. The diagnostic criteria for type 1 acute myocardial infarction were acute myocardial injury accompanied by clinical evidence of acute myocardial ischemia, at least one increase and/or decrease in cTn detected above the 99th percentile (the URL), and at least one of the following characteristics: symptoms of myocardial ischemia, new changes in the ischemic electrocardiogram, pathological Q-wave occurrence, or a time from onset to treatment of less than 6 h (Thygesen et al. 2018). The exclusion criteria for patients were as follows: under 18 years of age; lung diseases, such as inflammation, nodules or tumors; severe liver or kidney insufficiency; severe infection; rheumatic heart disease, primary cardiomyopathy, or other serious disease of the heart itself; recent ischemic stroke; severe immune system or blood system disease; long-term oral hormone use; and a history of malignant tumor. The study of human blood tissue was approved by the Ethics Committee of the Eighth Affiliated Hospital of Sun Yat-sen University (No. 202122-066-02) and complied with all relevant ethical guidelines. The participants with MI and healthy controls provided informed consent. There was no significant difference in basic information between the two groups (Supplementary Table 1).

### Animals

Male C57BL/6J mice (6–8 weeks old, weighing 16–20 g, and SPF grade) were purchased from Zhuhai BesTest Bio-Tech Co., Ltd. (China; Animal Quality Certificate No. 44822700019760). All animals were housed in a controlled environment at 22 °C with a 12-h light-dark cycle and provided ad libitum access to food and purified water. All animal experiments were approved by the Animal Ethics Committee of the Affiliated Eighth Hospital of Sun Yat-sen University (2022-066-01).

### Animal grouping and the MI model

The experimental mice were randomly divided into the sham-operated group (sham), MI group (MI + Vehicle), MI with low-dose (2.5 mg/kg) AubipyOMe group (MI + L), and MI with high-dose (5 mg/kg) AubipyOMe group (MI + H), with six mice in each group. The MI mouse model was induced by ligation of the left anterior descending (LAD) coronary artery as previously described (Zheng et al. 2013). In brief, anesthesia was induced using a mixture of 2% isoflurane and oxygen, followed by disinfection. Subsequently, a 1–2 cm incision was made in the fourth intercostal space to expose the heart, which was then extruded from the thoracic cavity and secured with 6 − 0 sutures to ligate the LAD. Successful occlusion was confirmed by an electrocardiogram with elevation of the ST segment (Weng et al. 2018). Subsequently, the mouse heart was repositioned in the thoracic cavity, and air and blood were expelled before the chest was closed. The sham group underwent the same procedure without ligation of the LAD artery. On Days 12, 15, and 18 after sham operation or LAD ligation surgery, mice in the MI with AubipyOMe group received intraperitoneal injections of different doses of AubipyOMe (Sigma‒Aldrich, USA, #SML2210) (Hu et al. 2022). The MI group received injections of PBS and DMSO (solvent for AubipyOMe). Four weeks after MI surgery, cardiac function was evaluated by ultrasound imaging. Subsequently, the mice were euthanized, and the ventricular portion of the heart sample and serum were collected for subsequent experiments.

### Echocardiography

The cardiac function of the mice was assessed using a high-resolution small animal ultrasound imaging system (Vevo2100, Visualsonics, Canada). Mice were initially anesthetized with 2% isoflurane using a mask. Measurements were taken for the following parameters: left ventricular (LV) internal diameter at end-systole (LVIDS) and end-diastole (LVIDD); interventricular septum thickness at end-diastole (IVSD) and end-systole (IVSS); and LV posterior wall thickness at end-diastole (LVPWD) and end-systole (LVPWS). Left ventricular fractional shortening (LVFS) and the left ventricular ejection fraction (LVEF) were calculated as described previously (Weng et al. 2018). The operators and analysts of the experiment were unaware of the group assignment.

### Histological staining

Paraffin sections of mouse heart samples were obtained by rapid dissection, fixation, embedding, and slicing. The Sect. (4 μm) were stained using Masson’s trichrome staining kit (Servicebio, China, #G1006) and Sirius Red Stain Kit (Servicebio, China, #G1472) following the manufacturer’s instructions. The percentage of fibrotic tissue was determined by measuring collagen deposition in the sections using ImageJ software. For immunofluorescence staining, the heart sections were deparaffinized and blocked with 10% donkey serum. Primary and secondary antibody staining was performed accordingly. Subsequently, nuclear staining was performed using DAPI. Positive results were quantified using ImageJ software. For immunofluorescence staining, the heart sections were deparaffinized, followed by blocking with 10% donkey serum. Primary (1:200; Cell Signaling Technology, USA, #19,245) and secondary(1:300; Servicebio, China, #GB21303) antibody staining was performed accordingly. Subsequently, nuclear staining was performed using DAPI. Six images were collected for each heart and quantitative analysis was performed by Image-J software. Relative positive rate (%) : (positive area/total area) *100%.

### Enzyme-linked immunosorbent assay (ELISA)

The Mouse ACP5 ELISA Kit was purchased from Signalway Antibody (USA, #EK12408), and the Human ACP5 ELISA Kit was purchased from BOSTER (China, #EK2138). ELISA kits were used to measure the levels of ACP5 in clinical patient samples and mouse blood samples according to the manufacturer’s instructions.

### Cell culture and transfection

CFs and cardiac myocytes (CMs) were obtained from neonatal mice aged 1–3 days according to previous methods (Medzikovic et al. 2021). Briefly, the collected ventricles were cut to approximately 1 mm3 and digested at 4 °C for 12 h with 0.25% trypsin solution (without EDTA) (Beyotime, China, #C0205). The supernatant was discarded after centrifugation. The tissue samples were digested in a 37 °C water bath with 0.1% type II collagenase (Solarbio, China, #C8150) for 10 min at a time until all tissues were digested. After centrifugation, the cell suspension was resuspended in high-glucose Dulbecco’s modified Eagle’s medium (GIBCO, USA, #C11995500BT) supplemented with 10% fetal bovine serum (GIBCO, USA, #16000-044) and 1% penicillin‒streptomycin (HyClone, USA, #SV30010). Fibroblasts were obtained after two consecutive attachment times (approximately 45 min each). CMs in the culture medium were transferred to another culture plate and incubated at 37 °C and 5% CO2. The cells were used in experiments when they were passaged for 3–4 generations. Small interfering RNA (siRNA) targeting ACP5 (siRNA-ACP5) and a siRNA-ACP5 negative control (si-NC) were synthesized by Ruibo Biotechnology (China). The ACP5 adeno-associated virus (rACP5) and the negative control adeno-associated virus (rNC) were synthesized by Hanbio (China). Transfection was performed according to the manufacturer’s instructions using a transfection reagent. The cells were incubated with 1 µM Ang II (Sigma‒Aldrich, USA, #A9525) for 24 h to induce CF activation. The Ro 67-7476 ERK activator (MCE, USA, #HY-100403) was used to activate the relevant signaling pathway.

### Immunofluorescence assay of cells

The cells were seeded into confocal dishes precoated with laminin (Sigma, USA, #L2020). After cell treatment, the cells were sequentially fixed, permeabilized, and blocked. After washing, the cells were incubated with primary antibody (ACP5, 1:200; Abcam; Britain, #ab191406) at 4 °C overnight. After washing, the cells were incubated with goat anti-rabbit IgG conjugated to Alexa 488 (1:500; Solarbio, China, #GB25303) for 45 min, and the cell nuclei were stained with DAPI at room temperature for 10 min. The cells were then observed with a confocal microscope (Primo Star, Zeiss Microsystems, Germany).

### EdU assay and CCK8 assay

The proliferation of CFs was determined by the CCK-8 method and EdU fluorescence staining. After cell intervention, the cells were incubated with CCK-8 reagent (10 µl per well) (Tongren, Japan, #CK04) in the dark for 2 h, and the absorbance was then measured at 450 nm. EdU fluorescence staining was performed to evaluate the proliferation of CFs according to the manufacturer’s instructions (Beyotime, China, #C0075). Positive cell rate: The ratio of the number of EdU positive cells to the total number of cells.

### Transwell assay and wound-healing assay

Cell migration ability was evaluated using wound-healing and Transwell assays according to previously described methods (Zhang et al. 2022). For the Transwell assay, a Transwell chamber (8-µm pore size) was placed in a 24-well plate containing culture medium. Fibroblasts (1.0 × 10^5 cells/mL) in serum-free medium were added to the Transwell chamber. After 24 h of incubation, the Transwell chambers were removed and gently washed with PBS, and the nonmigrated cells inside the chambers were removed by gently swabbing with a cotton swab. The chambers were then fixed with 4% paraformaldehyde for 15 min, washed with PBS, stained with crystal violet staining solution for 15 min, washed, and air-dried. Cell migration was observed under a microscope. In the wound-healing assay, when the density of CFs reached approximately 90% in a 6-well plate, a sterile pipette tip was used to scratch the cell layer. The cells were then gently washed with PBS to remove detached cells, and the medium was replaced with serum-free medium. Images of cell migration were captured at 0 and 24 h. The wound-healing and Transwell assays were analyzed by ImageJ software.

### Quantitative reverse transcription-polymerase chain reaction (qRT‒PCR)

Total RNA was extracted using TRIzol reagent (Sigma, USA, #T9424) following the standard protocol, and cDNA synthesis was subsequently performed using a reverse transcription kit (Accurate Biology, China, #AG11706). The SYBR Green Premix Pro Taq HS qPCR Kit (Accurate Biology, China, #AG11718) was used for qRT‒PCR on the Roche Diagnostic Light Cycler 480 System. The relative mRNA levels were calculated using the 2^(-ΔΔCt) method. The amplification was expected to be about 18–30 bp and the annealing temperature was 60℃. The specificity was validated using the National Center for Biotechnology Information(NCBI) BLAST after the primer design. The qRT-PCR primer sequences were as follows:

ACP5(F): 5ʹ-GGAACTTCCCCAGCCCTTAC-3ʹ,

ACP5(R): AGGTCTCGAGGCATTTTGGG;

COL1A1(F): 5ʹ-GAACTGGACTGTCCCAACCC-3ʹ,

COL1A1(R): 5ʹ-TTGGGTCCCTCGACTCCTAC-3ʹ;

α-SMA(F): 5ʹ-TCCACGAAACCACCTATAACAGC-3ʹ,

α-SMA(R): 5ʹ-CCAGACAGAGTACTTGCGTTCT-3ʹ;

CoL3(F): 5ʹ-GAGGAATGGGTGGCTATCCG-3ʹ ,

CoL3(F): 5ʹ-TCGTCCAGGTCTTCCTGACT-3ʹ;

β-actin(F): 5ʹ-ATGTGGATCAGCAAGCAGGA-3ʹ,

β-actin(R): 5ʹ-AAGGGTGTAAAACGCAGCTCA-3ʹ;

### Western blot analysis

CFs and cardiac tissue were lysed with RIPA lysis buffer (Beyotime, China, #P0013) containing phosphatase inhibitors (MCE, USA, #HY-K0021, #HY-K0022) and protease inhibitor cocktail (MCE, USA, #HY-K0010). The protein concentration was determined using a BCA Protein Assay Kit (CWBIO, China, #CW0014). The protein samples were mixed with SDS‒PAGE loading buffer (Beyotime, China, #P0015) and denatured at 100 °C for 10 min. After gel separation by SDS‒PAGE, the proteins were transferred onto PVDF membranes. Subsequently, the membranes were blocked with 5% skim milk at room temperature for 1 h. After washing, the membranes were incubated with the primary antibody overnight at 4 °C. On the following day, the washed membranes were incubated with an HRP-conjugated anti-rabbit IgG secondary antibody (1:5000; Cell Signaling Technology, USA, #7074). After washing, the chemiluminescent substrate was applied to the membranes, and the signal was detected using the ChemiDoc XRS + system (Bio-Rad). The grayscale values were analyzed using ImageJ software.

### Statistical analysis

All analyses were performed using GraphPad Prism 8.0.2 software (GraphPad Software Inc., La Jolla, CA, USA). The experiments were repeated at least three times. The data are presented as the mean ± standard deviation (mean ± SD) when they followed a normal distribution with equal variances. Otherwise, the data are reported as the median and interquartile range. Student’s t test was used to compare differences between two groups. One-way analysis of variance (ANOVA) was used for comparisons among multiple groups. A significance level of *P* < 0.05 was considered statistically significant.

## Results

### ACP5 expression is increased in MI patients, in vivo mouse fibrosis models, and in vitro mouse fibrosis models

**Fig. 1:**
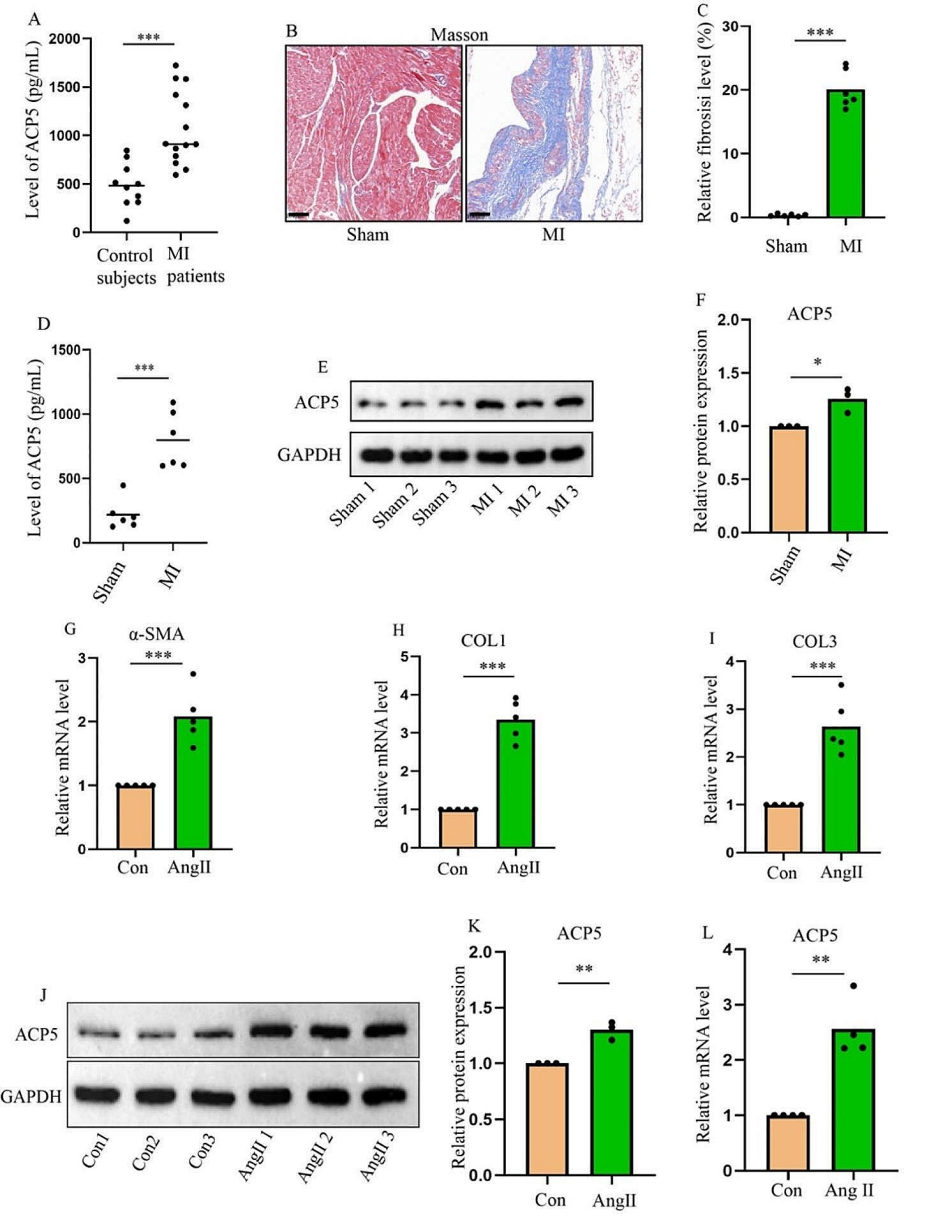
ACP5 expression is increased in patients with myocardial infarction, in vivo mouse models, and in vitro mouse models. (**A**) The expression of ACP5 in the blood of healthy individuals and postmyocardial infarction patients was detected by ELISA (control subjects: *n* = 10; MI patients: *n* = 14). (**B**-**C**) Masson staining and fibrosis ratio in each group (*n* = 6/group). Scale = 100 μm. (**D**) ACP5 expression in the blood of mice in different groups (*n* = 6/group). (**E**-**F**) Western blot analysis of ACP5 expression in the hearts of mice (*n* = 3/group). (**G**-**I**) Relative mRNA expression levels of α-SMA, COL1 and COL3 in CFs in different groups (*n* = 5/group). (**J**-**K**) CF Western blot analysis of ACP5 expression in different groups. (**L**) Relative mRNA expression levels of ACP5 in different CF groups (*n* = 4/group). *P < 0.05, **P < 0.01, and ***P < 0.001

Blood samples were collected from patients with clinical MI for ELISA detection, which demonstrated that ACP5 expression was increased after MI (Fig. 1A). Subsequently, the myocardial fibrosis model after MI was generated via ligation of the left anterior descending region. Figure 1B and C show that the myocardial fibrosis area of the mice increased at Day 28 after MI, indicating that the model was successfully established. Similarly, ACP5 expression was increased in the blood (Fig. 1D) and heart tissue (Fig. 1E, F) of MI mice. The protein expression of ACP5 in cardiomyocytes and CFs was detected to determine the distribution of ACP5. Because ACP5 was mainly distributed in CFs (Supplementary Fig. 1A, B), the present study focused on the role of ACP5 in CFs. Compared with those in the control group, the mRNA levels of α-SMA, COL1, and COL3 were significantly increased after Ang II treatment (Fig. 1G-I). Consistent with the results in heart tissue, qTR-PCR and Western blot analyses revealed increased ACP5 expression after Ang II treatment (Fig. 1J-L). Cellular immunofluorescence and quantitative analysis of ACP5 in CFs revealed that ACP5 expression increased after Ang II stimulation (Supplementary Fig. 1C, D). These results suggested that ACP5 expression is elevated during cardiac fibrosis after MI and is increased in vitro in Ang II-induced fibrosis models.

### Inhibition of ACP5 suppresses CF proliferation, migration, and transition into myofibroblasts

**Fig. 2:**
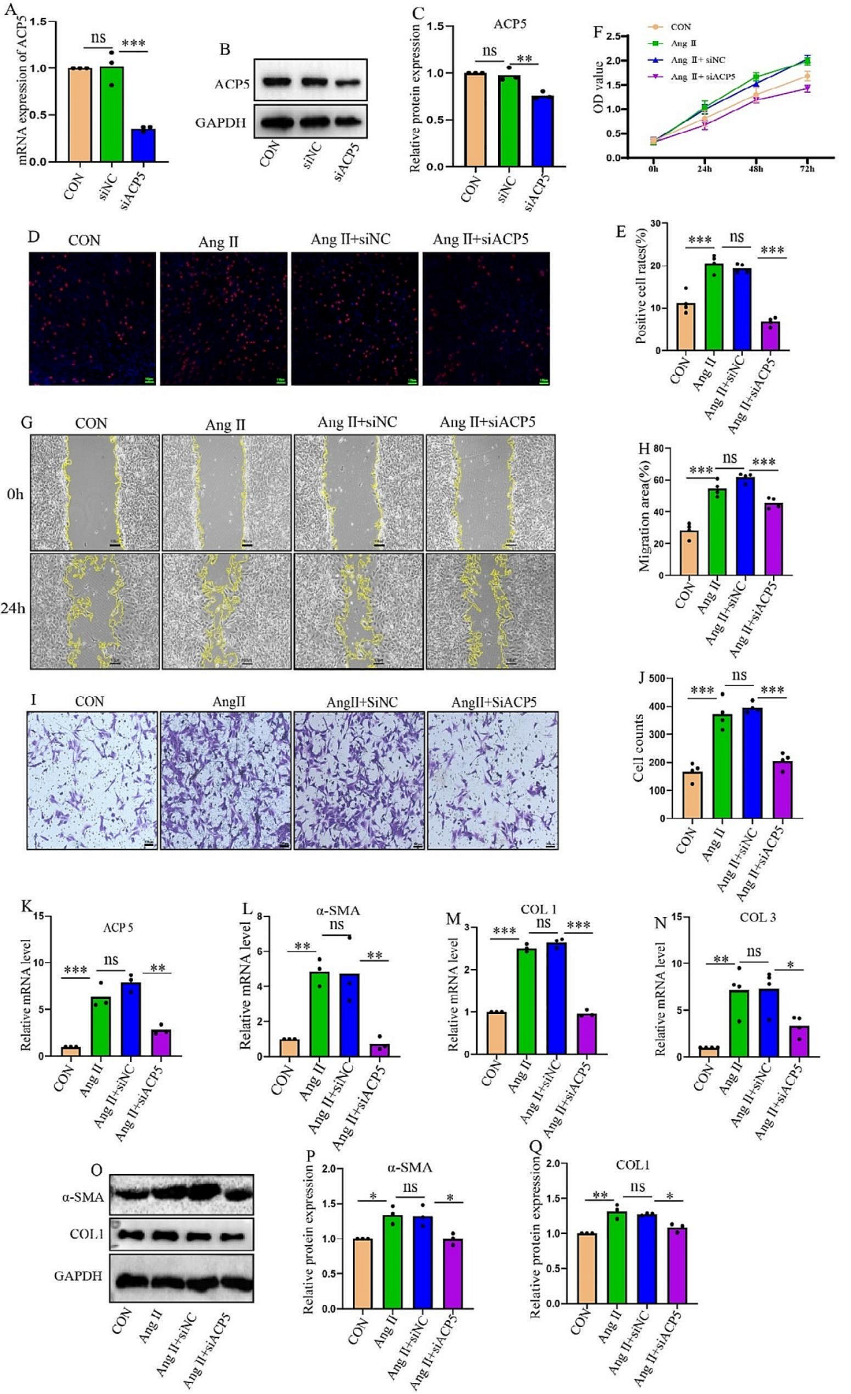
Inhibition of ACP5 suppresses CF proliferation, migration, and transition into myofibroblasts. (**A**) Relative mRNA expression levels after ACP5 silencing (*n* = 3/group). (**B**-**C**) Western blot analysis of the silencing efficiency of ACP5 (*n* = 3/group). (**D**-**E**) EdU assay was used to detect the cell proliferation rate of CFs (*n* = 4/group); scale bar = 100 μm. (**F**) OD values of CFs in different groups (*n* = 4/group). (**G**-**H**) Cell migration was assessed by a wound-healing assay (*n* = 4/group); scale bar = 100 μm. (**I**-**J**) Cell migration was assessed by Transwell assays (*n* = 4/group); scale bar = 100 μm. (**K**-**N**) Relative mRNA expression levels of ACP5, α-SMA, COL1, and COL3 in CFs in different groups (*n* ≥ 3/group). (**O**-**Q**) Western blot analysis of α-SMA and COL1 (*n* = 3/group). *P < 0.05, **P < 0.01, ***P < 0.001, and ns: *P* > 0.05

To suppress the endogenous expression of ACP5 in CFs, a small interfering RNA (siACP5) was constructed, and the efficiency of ACP5 inhibition was measured (Fig. 2A-C). EdU proliferation assays showed that after Ang II stimulation of CFs, the proliferation of CFs significantly increased, while inhibition of ACP5 decreased the number of proliferating cells (Fig. 2D, E). Consistent with these findings, the CCK-8 assay revealed that siACP5 significantly inhibited Ang II-induced CF proliferation (Fig. 2F). The effects of ACP5 on CF migration were observed by wound-healing and Transwell assays. As shown in Fig. 2G-J, Ang II promoted cell migration, while knockdown of ACP5 suppressed cell migration and delayed wound healing. Furthermore, qRT‒PCR demonstrated that Ang II stimulation increased the expression of ACP5, α-SMA, COL1, and COL3, and this increase was significantly reduced by silencing ACP5 (Fig. 2K‒N). Western blot analysis indicated that siACP5 inhibited the increase in collagen secretion induced by Ang II in CFs (Fig. 2O-Q). Together, these findings demonstrated that ACP5 inhibition suppresses Ang II-induced CF proliferation, migration, and phenotypic transition.

### Overexpression of ACP5 promotes CF proliferation, migration, and transition into myofibroblasts

**Fig. 3:**
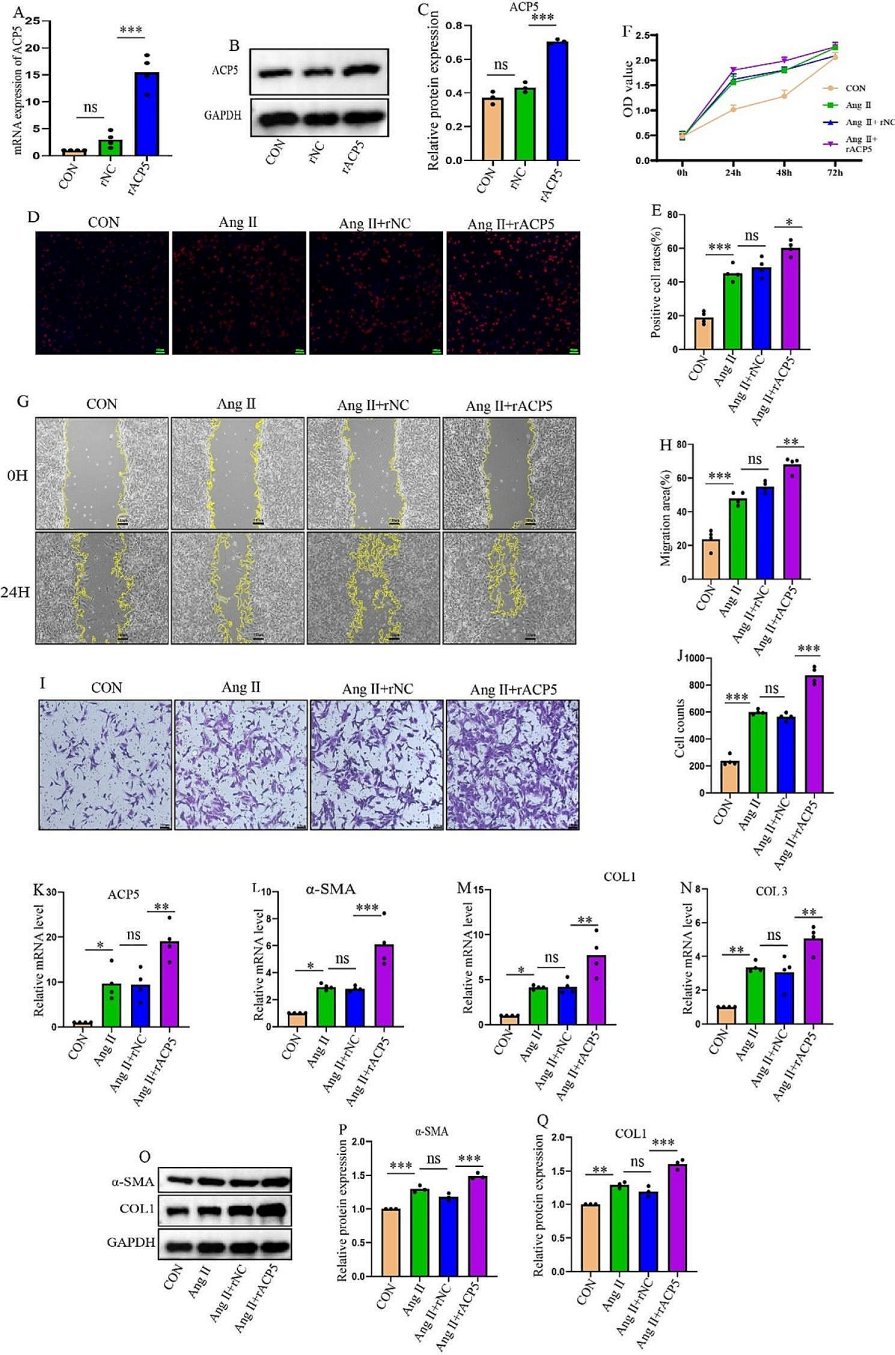
Overexpression of ACP5 promotes CF proliferation, migration, and transition into myofibroblasts. (**A**) Relative mRNA expression levels of ACP5 and the overexpression efficiency (*n* = 4/group). (**B**-**C**) Western blot analysis of the overexpression efficiency of ACP5 (*n* = 3/group). (**D**-**E**) The EdU assay was used to detect the cell proliferation rate of CFs (*n* = 4/group); scale bar = 100 μm. (**F**) OD values of CFs in different groups (*n* = 3/group). (**G**-**H**) Cell migration was assessed by a wound-healing assay (*n* = 4/group); scale bar = 100 μm. (**I**-**J**) Cell migration was assessed by Transwell assays (*n* = 4/group); scale bar = 100 μm. (**K**-**N**) Relative mRNA expression levels of ACP5, α-SMA, COL1, and COL3 in CFs in different groups (*n* = 4/group). (**O**-**Q**) Western blot analysis of α-SMA and COL1 (*n* = 3/group). *P < 0.05, **P < 0.01, ***P < 0.001, and ns: *P* > 0.05

To investigate the role of ACP5 in cardiac remodeling, the impact of ACP5 overexpression on CF proliferation, migration, and collagen synthesis was evaluated. First, ACP5 overexpression in CFs was confirmed (Fig. 3A-C). EdU staining indicated a significant increase in CF proliferation after overexpressing ACP5 under Ang II stimulation (Fig. 3D-E). The CCK-8 assay demonstrated greater cell viability in the Ang II + ACP5 overexpression group than in the Ang II + rNC group (Fig. 3F). Furthermore, the wound-healing and Transwell assays demonstrated that ACP5 overexpression augmented CF migration (Fig. 3G-J). In addition, qRT‒PCR showed that ACP5 overexpression in CFs significantly upregulated the mRNA expression of fibrosis-related factors (Fig. 3K‒N). Consistent with these findings, ACP5 overexpression increased the protein expression levels of A-SMA and COL1 in CFs (Fig. 3O and Q). These findings indicated that elevated ACP5 expression facilitates CF proliferation, migration, and differentiation.

### ACP5 inhibition suppresses fibrosis in MI mice and enhances cardiac function

**Fig. 4:**
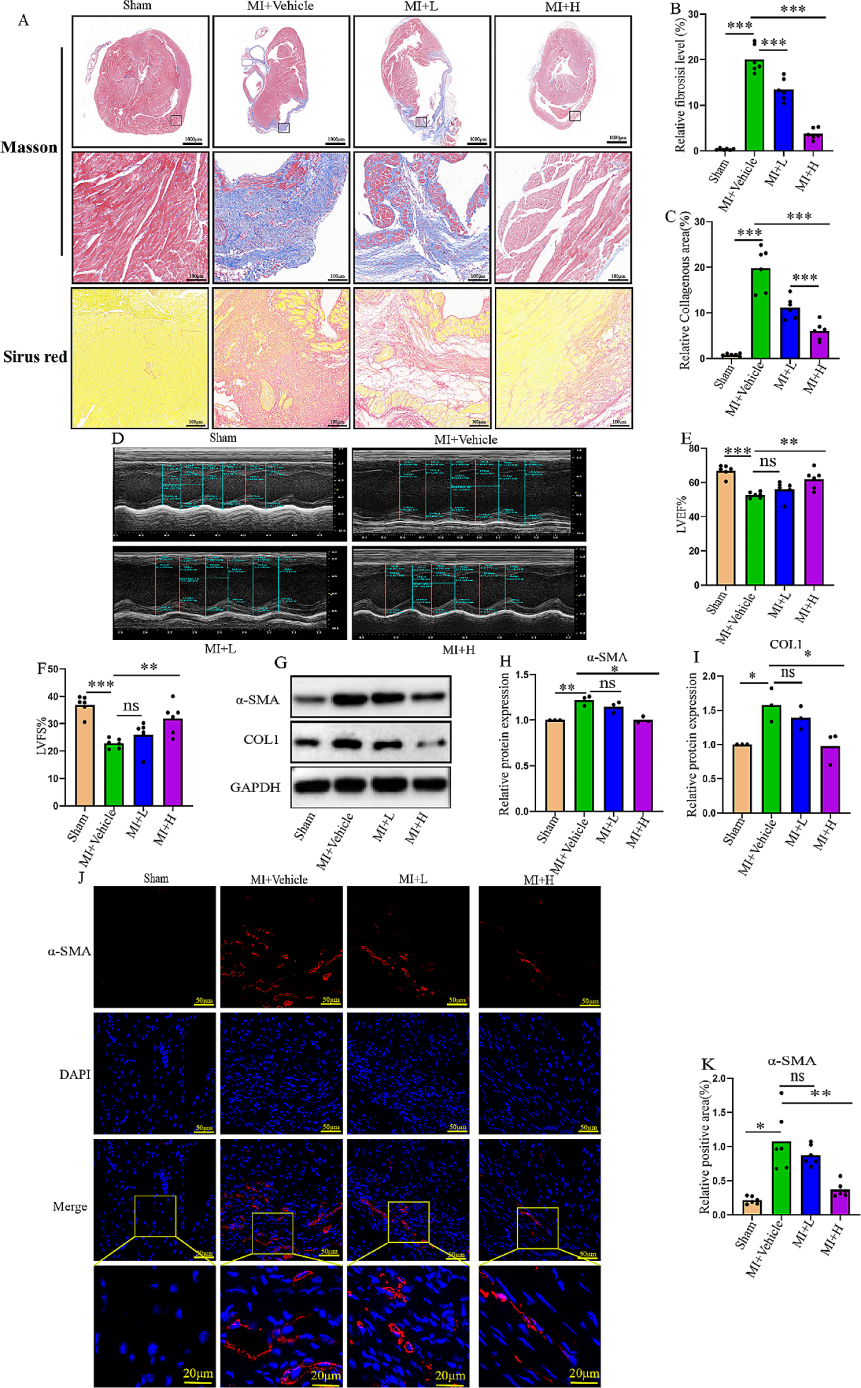
ACP5 inhibition suppresses fibrosis in MI mice and enhances cardiac function. (**A**) Masson staining and Sirius red staining in each group (*n* = 6/group; upper layer, scale bar = 1000 μm; middle layer, scale bar = 100 μm; lower layer, scale bar = 100 μm). (**B**) Masson staining of fibrosis. (**C**) Sirius red staining of the collagen area. (**D**) Representative images of echocardiography. (**E**-**F**) Echocardiographic measurements of LVEF (E) and LVFS (F) (*n* = 6/group). (**G**-**I**) Western blot analysis of α-SMA and COL1 in the hearts of mice in different groups (*n* = 3/group). (**J**-**K**) Immunofluorescence staining of α-SMA in the hearts of mice in different groups (*n* = 4/group); scale bar = 50 μm. (The bottom-layer image in Fig. 4J is the locally enlarged image circled in the Merge graph, bar = 20 μm). *P < 0.05, **P < 0.01, ***P < 0.001, and ns: *P* > 0.05

To investigate the role of ACP5 in myocardial fibrosis, the ACP5 inhibitor, AubipyOMe, was administered to mice after MI. Masson and Sirius red staining showed increased collagen in the area of heart infarction (Masson stain: blue; Sirius red staining: red). In the high-dose AubipyOMe group, the fibrosis area and collagen deposition in the infarction area were significantly reduced (Fig. 4A-C). Ultrasound revealed that the high dose significantly increased the LVEF and LVFS of the mouse heart, as well as restored cardiac function to a certain extent (Fig. 4D-F). Additionally, Western blot analysis confirmed that the expression levels of α-SMA and COL1 were upregulated in mice with MI, but the ACP5 inhibitor counteracted these fibrotic effects (Fig. 4G-I). α-SMA is a representative marker of the activation of CFs into myofibroblasts. Immunofluorescence staining revealed increased α-SMA expression in the infarct area of the heart after MI, which was reduced in the high-dose group treated with AubipyOMe (Fig. 4J, K). In conclusion, the inhibition of ACP5 blocks fibroblast activation, reduces cardiac fibrosis in the infarction area, and improves cardiac function.

### ACP5 affects the GSK3β/β-catenin signal transduction pathway

**Fig. 5:**
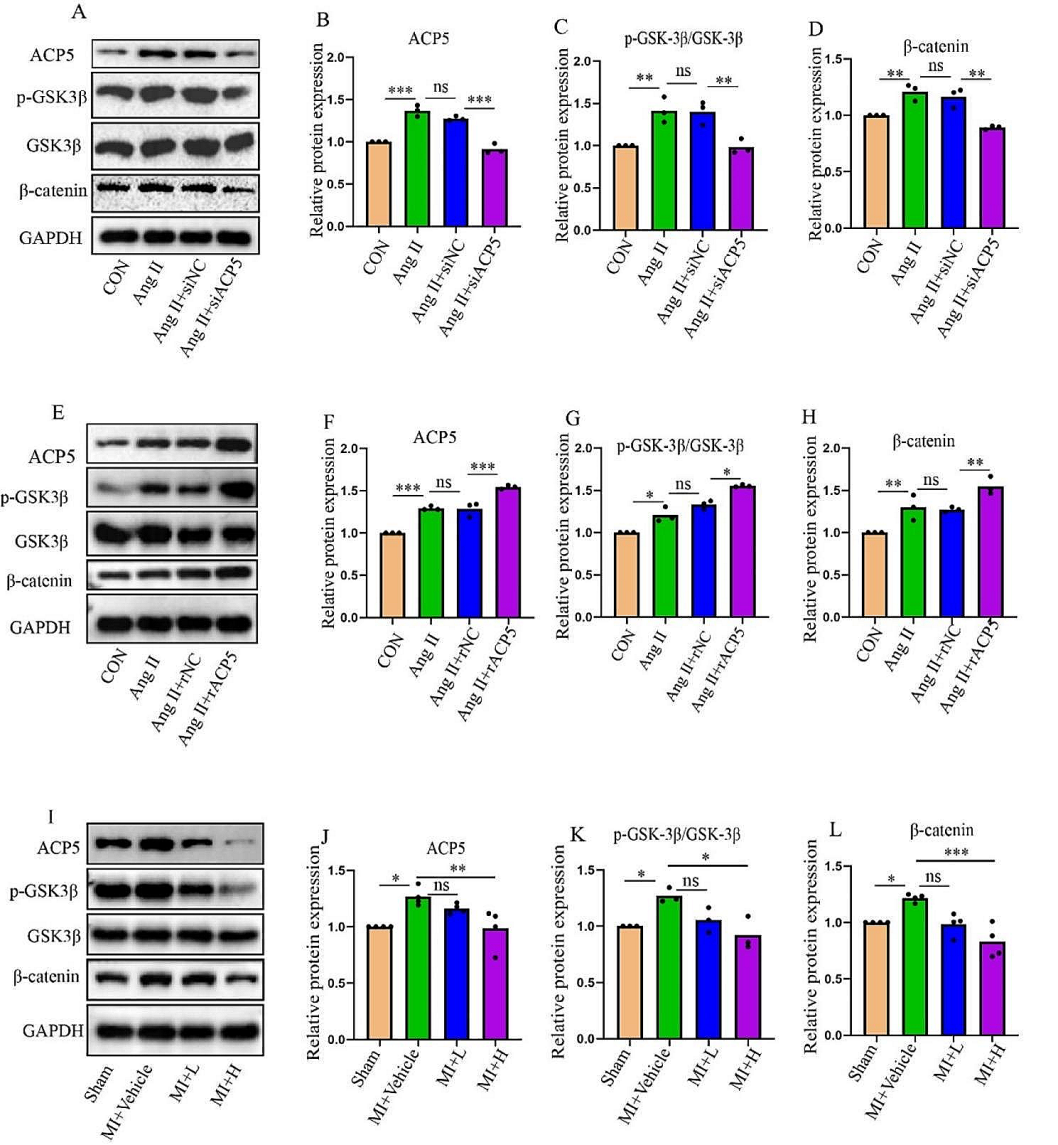
ACP5 affects the GSK3β/β-catenin signal transduction pathway. (**A**-**D**) Western blot analysis of the expression levels of ACP5, p-GSK3β, GSK3β, and β-catenin in ACP5-deficient CFs (*n* = 3/group). (**E**-**H**) Western blot analysis of the expression levels of ACP5, p-GSK3β, GSK3β, and β-catenin in CFs overexpressing ACP5 (*n* = 3/group). (**I**-**L**) Western blot analysis of the expression levels of ACP5, p-GSK3β, GSK3β, and β-catenin in the hearts of mice in different groups (*n* ≥ 3/group). *P < 0.05, **P < 0.01, ***P < 0.001, and ns: *P* > 0.05

Because ACP5 inhibition alleviated cardiac fibrosis in mice with MI Next, the underlying mechanisms by which ACP5 functions were further explored. GSK3β/β-catenin plays an important role in diseases associated with cardiac fibrosis (Daskalopoulos and Blankesteijn 2021). Therefore, the present study investigated whether ACP5 modulates the GSK3β/β-catenin signaling pathway. The GSK3β/β-catenin pathway was activated in CFs after Ang II stimulation, but ACP5 knockout reduced the expression of GSK3β/β-catenin (Fig. 5A-D). In contrast, ACP5 overexpression activated the GSK3β/β-catenin pathway (Fig. 5E-H). Moreover, the expression of GSK3β/β-catenin was increased in the heart tissue of the MI model in vivo; however, this change was inhibited by high doses of the ACP5 inhibitor (Fig. 5I-L). These results suggested that ACP5 induces fibrotic responses in cells by influencing the GSK3β/β-catenin pathway.

### ACP5 affects CF activation by regulating the ERK/GSK3β/β-catenin pathway

**Fig. 6:**
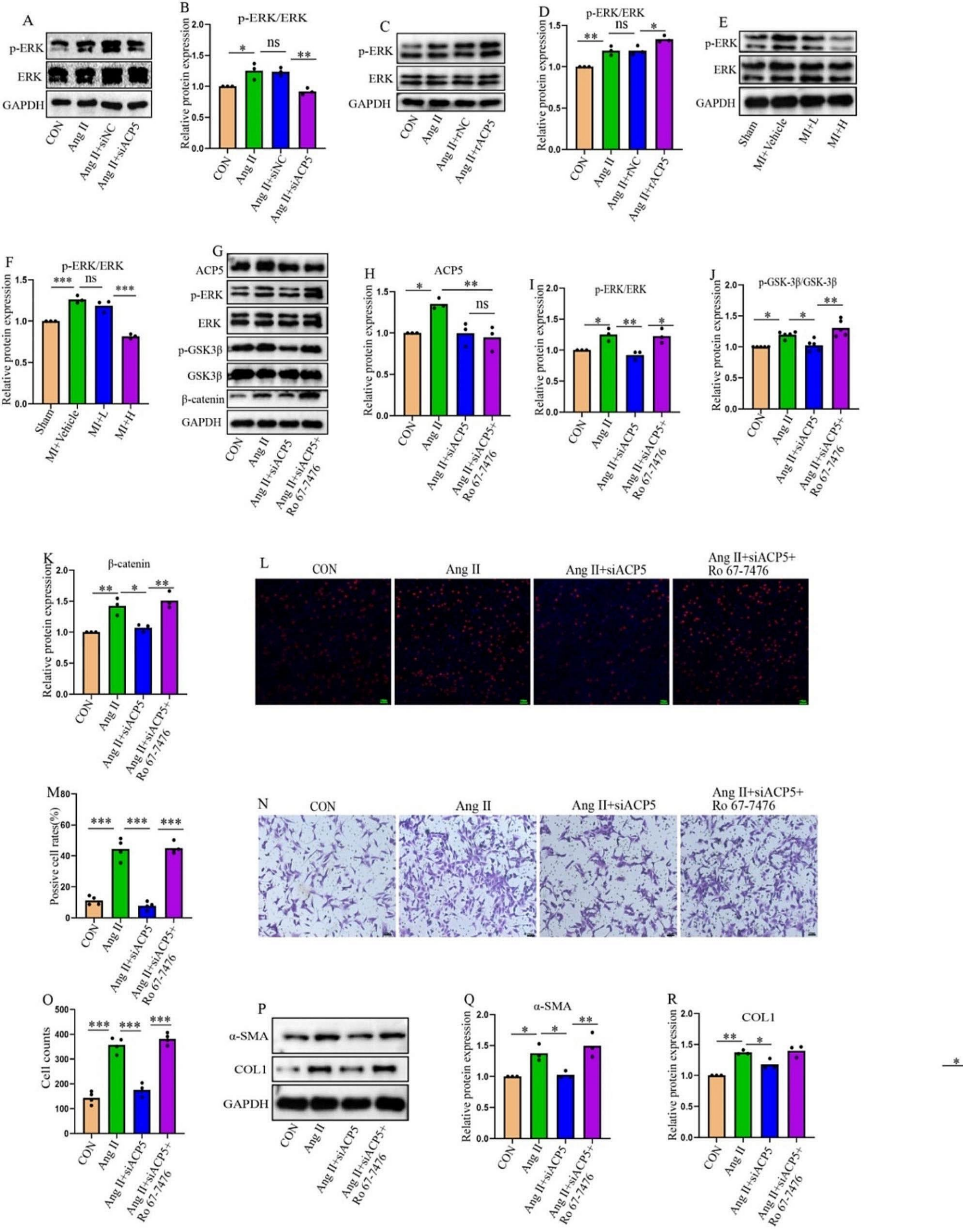
ACP5 affects CF activation by regulating ERK. (**A**-**B**) Western blot analysis of the expression levels of p-ERK and ERK in ACP5-deficient CFs (*n* = 3/group). (**C**-**D**) Western blot analysis of the expression levels of p-ERK and ERK in CFs overexpressing ACP5 (*n* = 3/group). (**E**-**F**) Western blot analysis of the expression levels of p-ERK and ERK in the hearts of mice in different groups (*n* = 3/group). (**G**-**K**) Western blot analysis of the expression levels of ACP5, p-ERK, ERK, p-GSK3β, GSK3β, and β-catenin in CFs pretreated with Ro 67-7476 (an ERK agonist) (*n* = 3/group). (**L**-**M**) The EdU assay was used to detect the proliferation rate of CFs pretreated with Ro 67-7476 (an ERK agonist) (*n* = 4/group); scale bar = 100 μm. (**N**-**O**) Cell migration was assessed by Transwell assays (*n* = 4/group); scale bar = 100 μm. (**P**-**R**) Western blot analysis of α-SMA and COL1 in the hearts of mice in different groups (*n* = 3/group).*P < 0.05, **P < 0.01, ***P < 0.001, and ns: *P* > 0.05

Recent studies have shown that ERK activation is related to the inactivation of GSK3β (Nagarajan et al. 2012). Considering that ERK plays an important role in cardiac fibrosis, the effect of ACP5 on ERK and its relationship with GSK3β/β-catenin were explored. After Ang II stimulation, the level of phosphorylated ERK increased, but the level of phosphorylated ERK decreased when ACP5 was inhibited (Fig. 6A, B). Consistent with this finding, overexpression of ACP5 promoted the activation of ERK (Fig. 6C, D). In vivo experiments revealed that the level of ERK phosphorylation increased significantly in the model group (Fig. 6E, F). To determine the critical role of ERK in ACP5-mediated cell damage via the GSK3β/β-catenin pathway, a specific ERK activator (Ro 67-7476) was used in the presence of ACP5 knockout. Figure 6G and H show that ERK activation had no effect on ACP5. However, the ERK activator reversed the decrease in GSK3β/β-catenin expression induced by ACP5 deficiency (Fig. 6G-K). Consistent with these findings, cell proliferation, migration, and phenotypic transformation were inhibited by ACP5 deficiency, while the ERK activator inhibited CF activation (Fig. 6L-R). These results suggested that ACP5 may promote CF activation by influencing the ERK/GSK3β/β-catenin signaling pathway, thereby leading to cardiac fibrosis.

## Discussion

In patients with MI, excessive remodeling of myocardial fibers can initiate a chain of events leading to myocardial fibrosis and heart failure (Zhang et al. 2023). Current clinical use of drugs, such as angiotensin-converting enzyme (ACE) inhibitors, beta-blockers, and aldosterone antagonists, is hampered by side effects, such as hypotension and bradycardia (Lin et al. 2023). Therefore, there is an urgent need to study the potential mechanism of myocardial fibrosis after MI and to identify new pharmacological targets to intervene in or delay the process of cardiac fibrosis.

In recent years, ACP5 has received increasing attention. Hu et al. demonstrated that increased ACP5 expression significantly enhances cell proliferation, migration, invasion, and epithelial–mesenchymal transition (EMT) (Hu et al. 2020). Some studies have shown that some specific factors inhibiting CFs inhibit the proliferation and migration of CFs in primary mice, the differentiation of CFs into myofibroblasts, and the development of cardiac fibrosis (Subbaiah et al. 2023). Additionally, Bai et al. reported that miR-877 alters cell viability, invasion, and EMT by regulating ACP5 (Bai et al. 2020). Jesper et al. constructed a bleomycin-induced pulmonary fibrosis model and reported reduced cytokine production and lung injury in ACP5 knockout mice (Bergwik et al. 2024). However, the role of ACP5 in cardiovascular diseases remains unclear. The present study demonstrated that ACP5 expression is upregulated in fibrotic cardiac tissues and that inhibiting ACP5 reduces the extent of post-MI cardiac fibrosis. Furthermore, the present study demonstrated that ACP5 promotes the proliferation, migration, and phenotypic transformation of CFs. Although studies have shown differences in ACP5 protein levels in CFs, other cells and effectors, such as extracellular vesicles (Morelli et al. 2019) and non-coding RNAs (Castillo-Casas et al. 2023; Jankauskas et al. 2023), cannot be ruled out.

Glycogen synthase kinase-3 (GSK-3) is a serine/threonine kinase composed of two isoforms, namely, α and β. The Wnt/β-catenin signaling pathway is well known to play a crucial role in stimulating cell proliferation, differentiation, and migration, and it is indispensable for the development of cardiac fibrosis (Tao et al. 2016). During the quiescent phase, GSK3β-mediated phosphorylation maintains low β-catenin levels, and β-catenin is targeted for ubiquitination and proteasomal degradation (Guo et al. 2017). These findings demonstrate the important role of GSK3β in maintaining β-catenin homeostasis in cells during cardiac fibrosis and suggest that the GSK3β/β-catenin signaling pathway represents a therapeutic target for treating myocardial fibrosis. The present study revealed that when ACP5 is overexpressed or inhibited, the phosphorylated form of GSK3β and the expression of β-catenin are activated or inhibited, respectively. Although previous studies have shown that Ang II activates the Wnt/β-catenin signaling pathway (Czepiel et al. 2022), the present findings indicated that ACP5 plays an important regulatory role in GSK3β/β-catenin signaling.

**Fig. 7:**
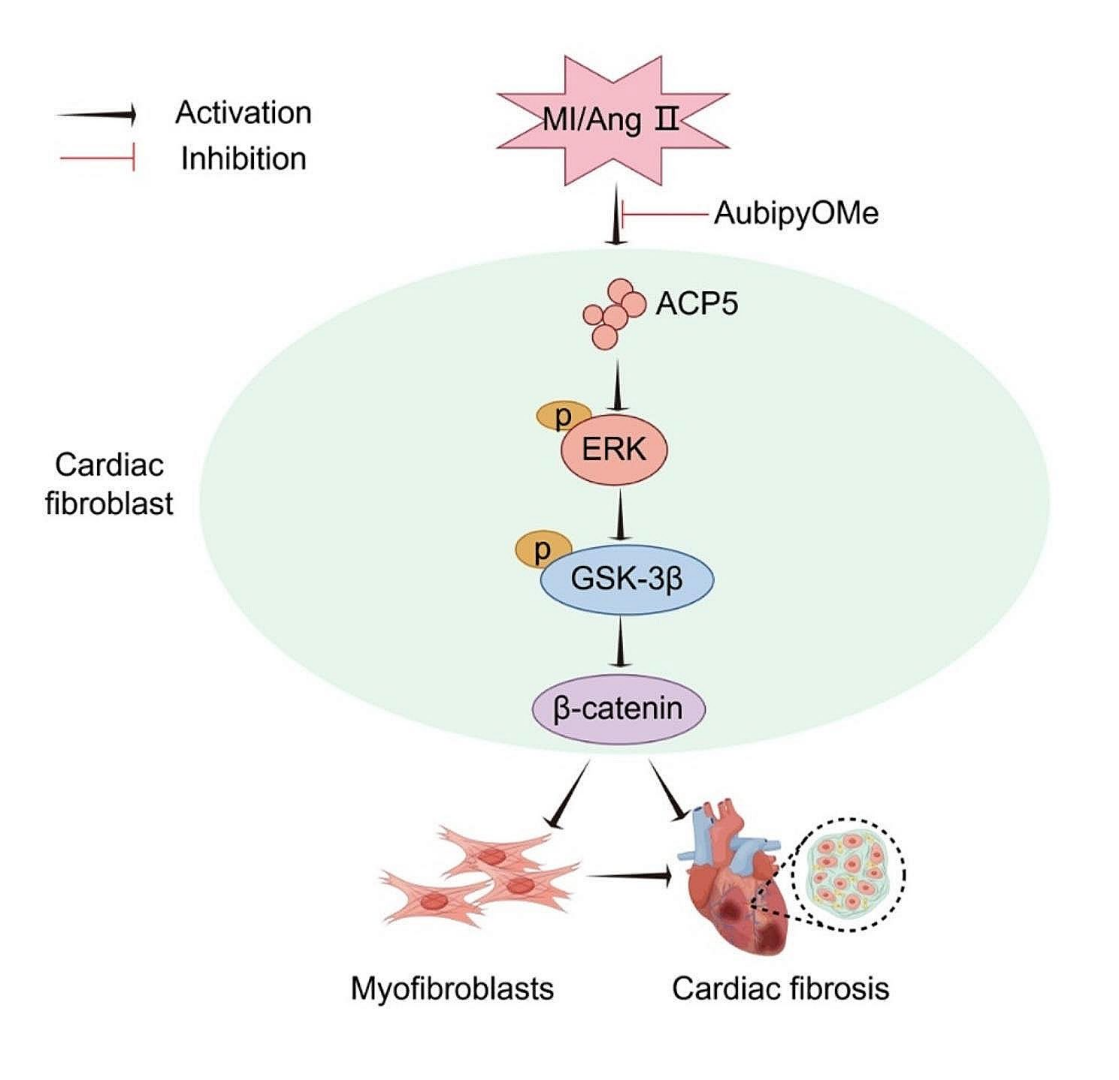
Schematic diagram of the mechanism of ACP5 in myocardial fibrosis after MI. Under the stimulation of MI or Ang II, the increased expression of ACP5 activates the ERK/GSK3β/β-catenin signaling pathway, which promotes the transformation of CFs into myofibroblasts with more active proliferation, migration and fibrosis, leading to the onset of myocardial fibrosis. (Generated by Figdraw)

As a member of the MAPK signaling transduction enzyme superfamily, ERK is an important signaling molecule that regulates cell growth, proliferation, and differentiation, and it is also involved in myocardial hypertrophy and fibrosis (Hu et al. 2016; Tian et al. 2021; Wang et al. 2021). He et al. reported that ACP5 increases the level of phosphorylated ERK, thereby promoting the progression of lung cancer (He et al. 2019). Therefore, the present study investigated whether ACP5 affects GSK3β/β-catenin through ERK. The inhibitory effect of ACP5 on GSK3β/β-catenin was altered by an activator of ERK, thereby suggesting a relationship between the activation of ERK and the inactivation of GSK3β/β-catenin. Other studies have reported similar findings. Nagarajan et al. demonstrated that the activation of ERK1/2 leads to the phosphorylation and inactivation of GSK3β, while the inhibition of ERK suppresses GSK3β phosphorylation in lung fibroblasts (Nagarajan et al. 2012). Caraci et al. reported that the activation of ERK1/2 induced by specific factors targets the GSK3β/β-catenin pathway, transforming human lung fibroblasts into myofibroblasts (Caraci et al. 2008). Together, these results suggest that ACP5 may regulate CF proliferation, migration, and phenotypic transition by influencing ERK phosphorylation and subsequently triggering the GSK3β/β-catenin pathway, leading to cardiac fibrosis (Fig. 7). This is the first study to propose that ACP5 may promote myocardial fibrosis after MI through the regulation of the ERK/GSK3β/β-catenin pathway.

The present study revealed two important findings. First, ACP5 plays a crucial role in regulating myocardial fibrosis following MI. Second, ACP5 affects the proliferation, migration, and phenotypic transformation of fibroblasts by modulating the activity of the ERK/GSK3β/β-catenin signaling pathway. The present results support the hypothesis that ACP5 plays a key role in the pathogenesis of cardiac fibrosis after MI and provide insights into the regulatory mechanisms of myocardial fibrosis. Therefore, targeting ACP5 may be a potential therapeutic approach for treating MI and offers new research directions for the treatment of myocardial fibrosis following MI.

In the present study, the bias caused by researchers’ expectations was reduced through standardized experimental procedures and a double-blinded experimental design. However, the present study had several limitations. The present study only detected the expression of ACP5 in blood samples from clinical patients after admission to the hospital for myocardial infarction. A larger cohort of patients is needed to further study the changes in ACP5 expression over time after MI. In addition, an ACP5 inhibitor, which was systemically introduced without specificity, was used to explore the role of ACP5 in vivo. Future studies will utilize ACP5 conditional knockout mice as an in vivo model.

## Conclusions

ACP5 influences the proliferation, migration, and phenotypic transition of CFs via modulating the ERK/GSK3β/β-catenin signaling pathway, leading to the development of myocardial fibrosis after MI.

### Electronic supplementary material

Below is the link to the electronic supplementary material.

Supplementary Material 1

Supplementary Material 2

Supplementary Material 3

Supplementary Material 4: Fig. 1 Western blot analysis of the ACP5 expression in cardiomyocytes and CFs (A-B). Immunofluorescence expression and quantitative analysis of ACP5 in CFs (C-D). **P < 0.01. table 1 Clinical information baseline characteristics of clinical subjects.

## Data Availability

The data supporting the findings of this study are available from the corresponding authorupon reasonable request.
