# Supplementary material for: Inhibition of tartrate-resistant acid phosphatase 5 can prevent cardiac fibrosis after myocardial infarction
Source: Mol Med. 2024 Jun 15;30:89. doi: 10.1186/s10020-024-00856-1 (PMC11179352; doi:10.1186/s10020-024-00856-1)
Supplement: Supplementary file 1 — Supplementary Material 1 [file 10020_2024_856_MOESM1_ESM.pdf]

SPRINGER NATURE

Author Services

Editing Certificate

This document certifies that the manuscript

Inhibition of ACP5 can prevent cardiac fibrosis after myocardial infarction

prepared by the authors

Shujun Yang,1, Liying Pei,1, Zijie Huang,1, Yinsheng Zhong, Jun Li, Yinghui Hong, Huibao Long, Xuxiang Chen, Changqing Zhou, Guanghui Zheng, Chaotao Zeng, Haidong Wu, Tong Wang, *

was edited for proper English language, grammar, punctuation, spelling, and overall style by one or more of the highly qualified native English speaking editors at SNAS.

This certificate was issued on **May 13, 2024** and may be verified on the SNAS website using the verification code **5BA9-2496-D51F-D417-5D67**.

Neither the research content nor the authors' intentions were altered in any way during the editing process. Documents receiving this certification should be English-ready for publication; however, the author has the ability to accept or reject our suggestions and changes. To verify the final SNAS edited version, please visit our verification page at secure.authorservices.springernature.com/certificate/verify. If you have any questions or concerns about this edited document, please contact SNAS at support@as.springernature.com.

SNAS provides a range of editing, translation, and manuscript services for researchers and publishers around the world. For more information about our company, services, and partner discounts, please visit authorservices.springernature.com.
