# Supplementary material for: Inhibition of tartrate-resistant acid phosphatase 5 can prevent cardiac fibrosis after myocardial infarction
Source: Mol Med. 2024 Jun 15;30:89. doi: 10.1186/s10020-024-00856-1 (PMC11179352; doi:10.1186/s10020-024-00856-1)

original western blot for three repeats in figure 2B and 2O

2B

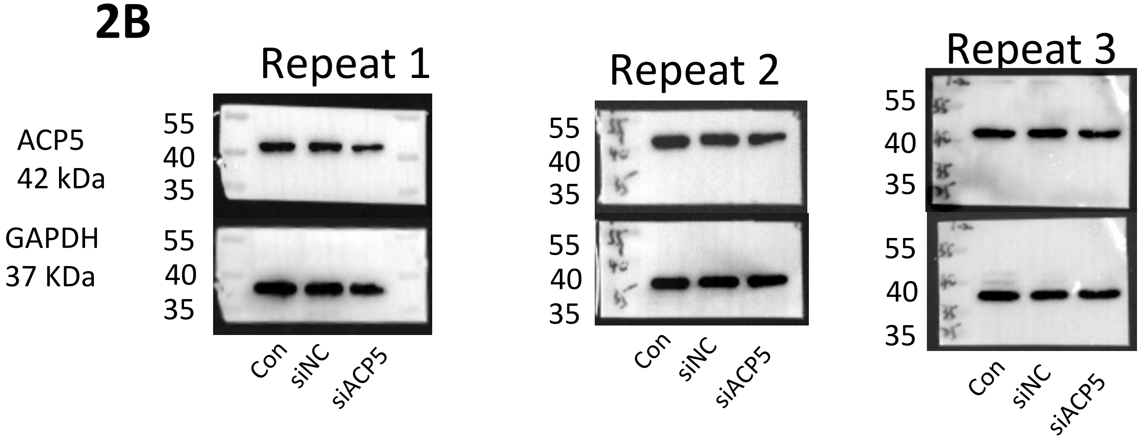

2O

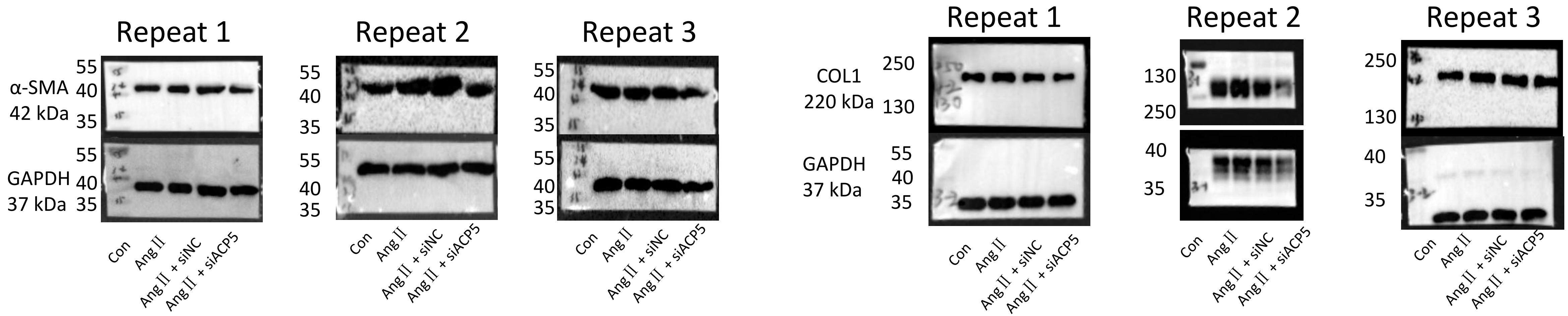

original western blot for three repeats in figure 3B and 3O

3B

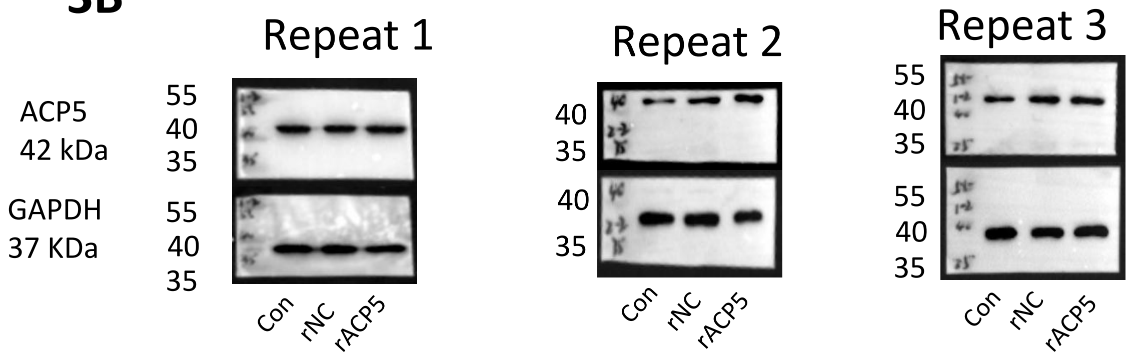

3O

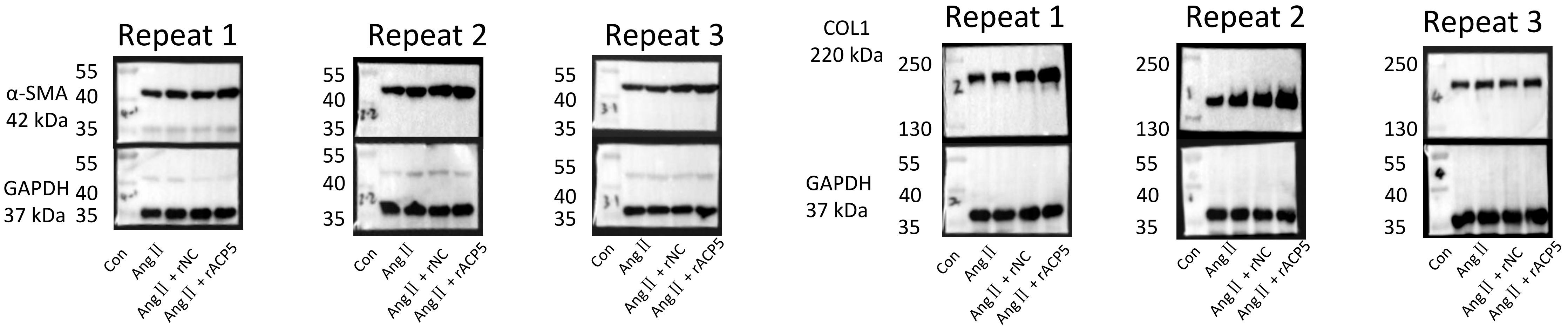

original western blot for three repeats in figure 4F

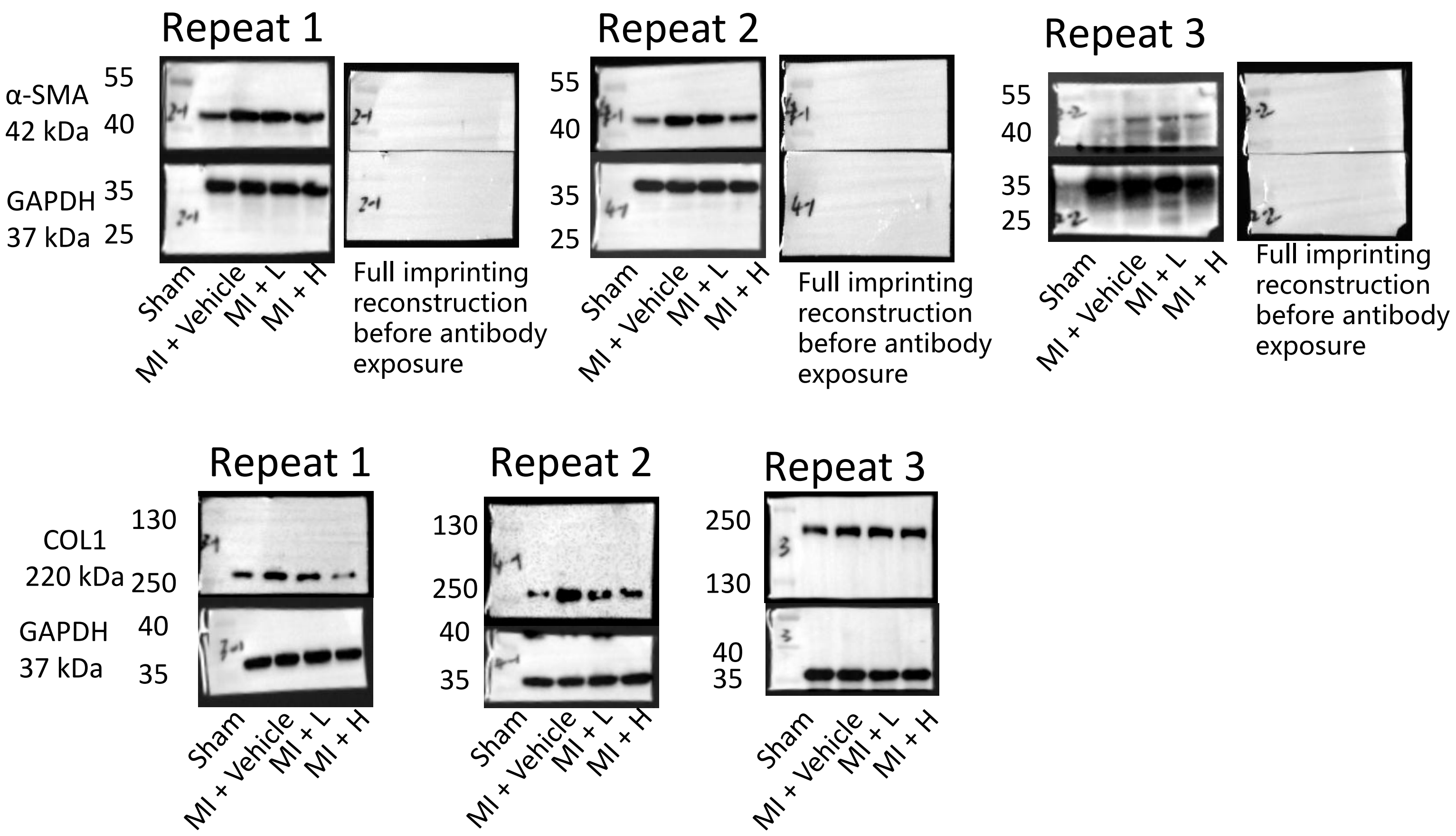

original western blot for three repeats in figure 5A

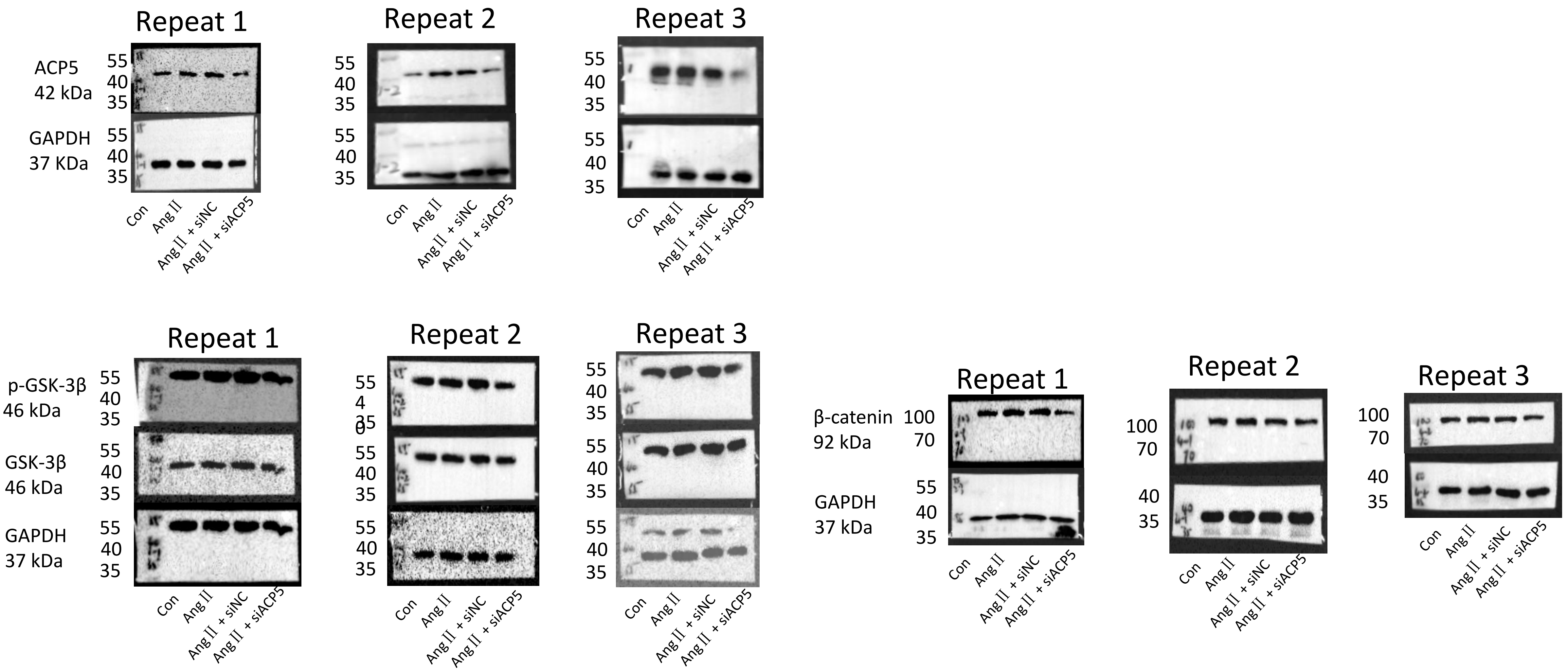

original western blot for three repeats in figure 5E

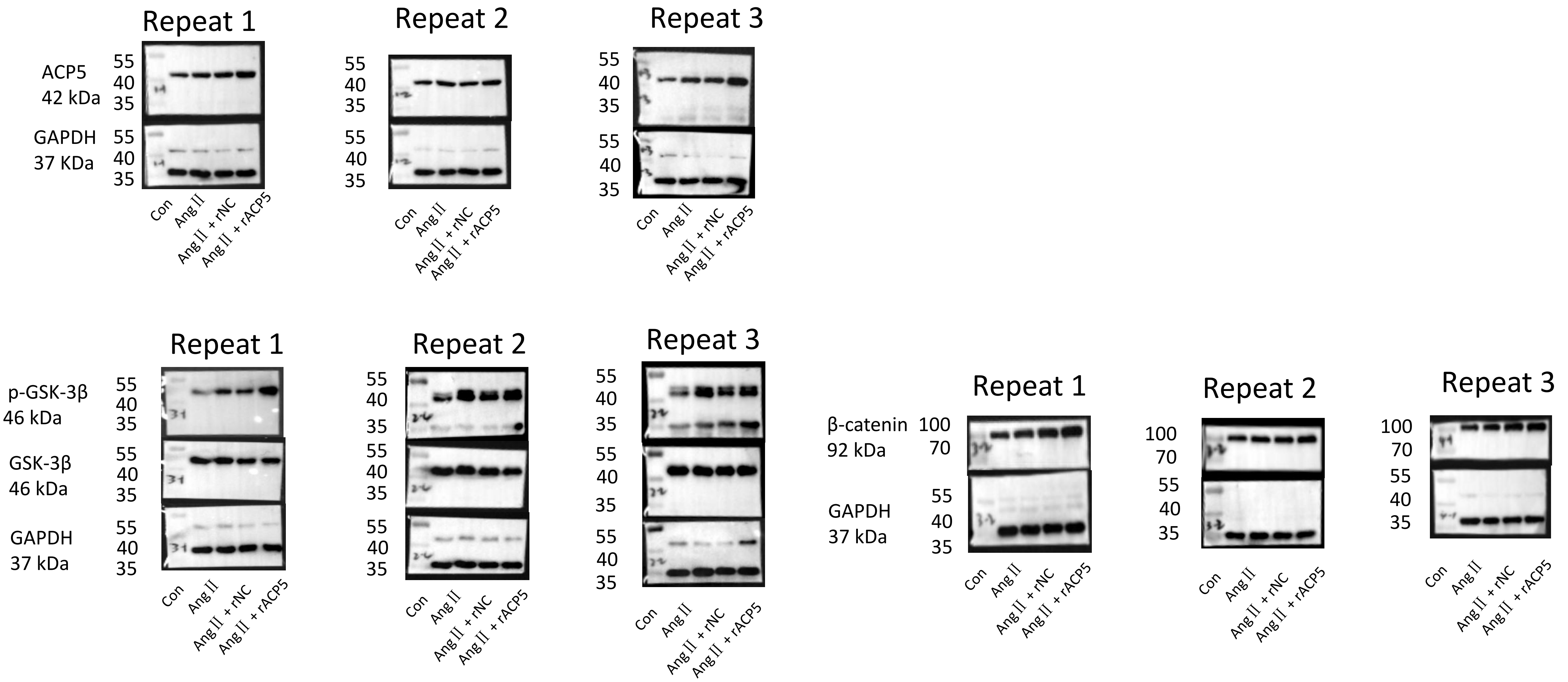

original western blot for three repeats in figure 5I

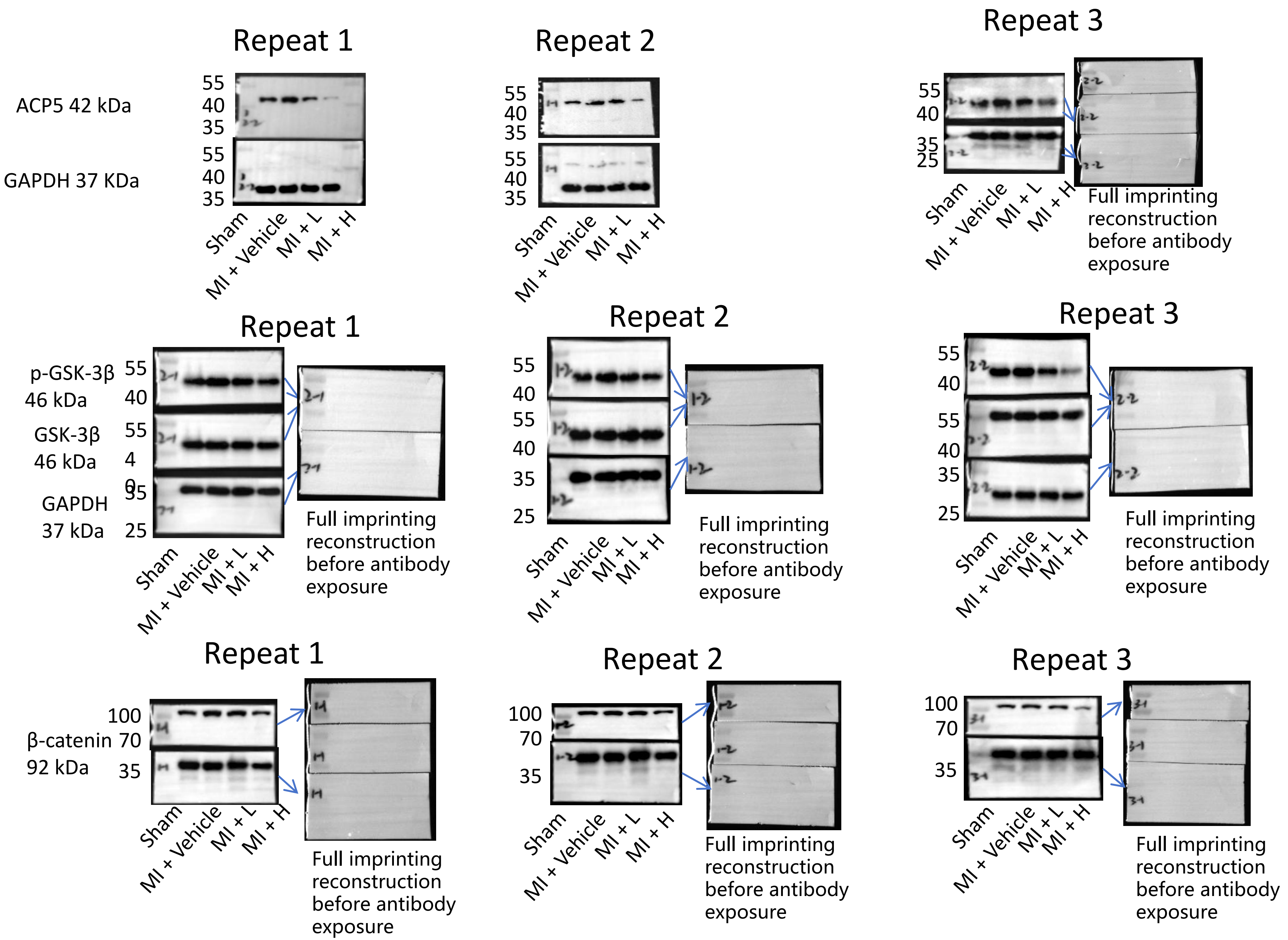

original western blot for three repeats in figure 6A,C and E

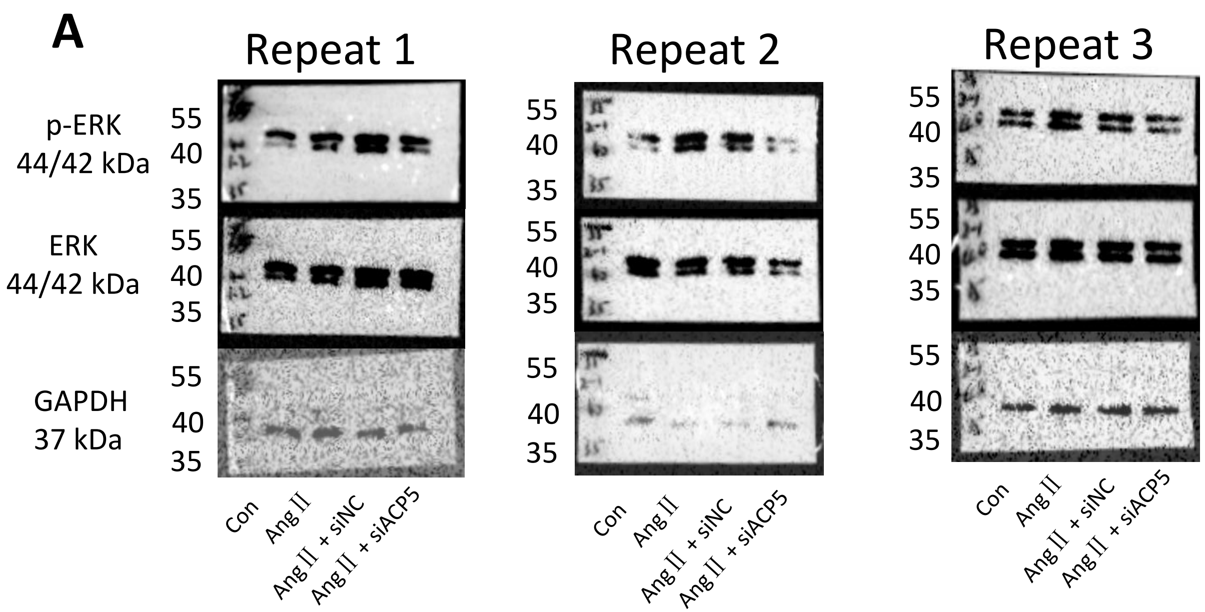

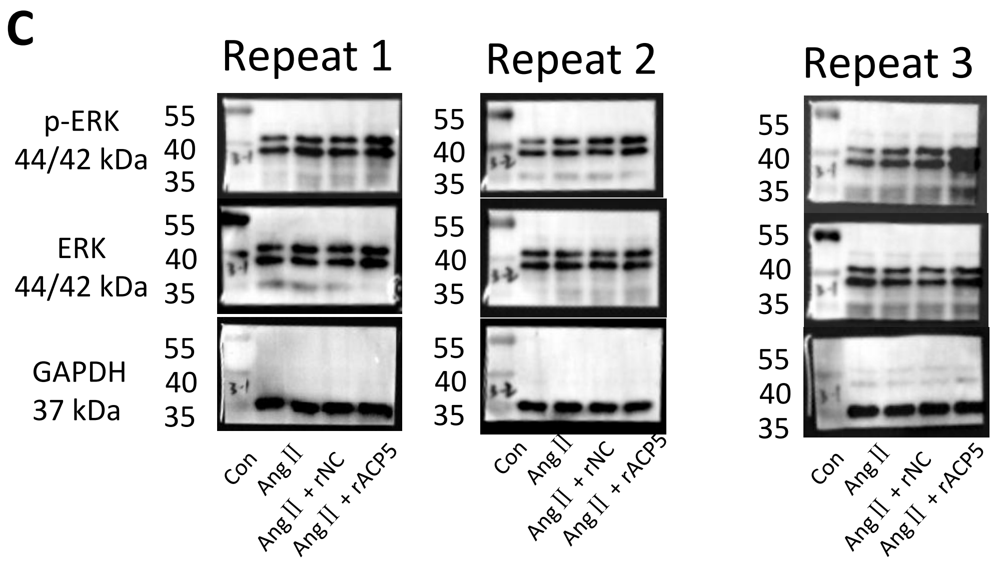

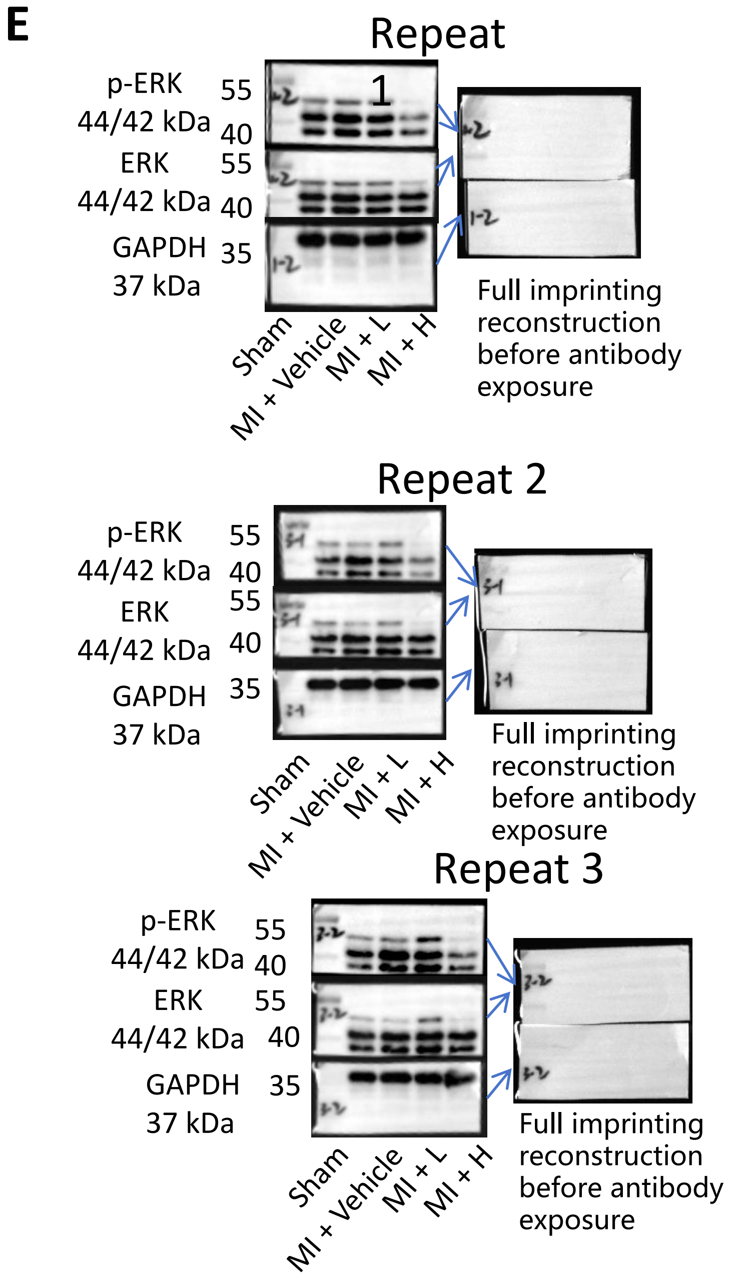

original western blot for three repeats in figure 6G

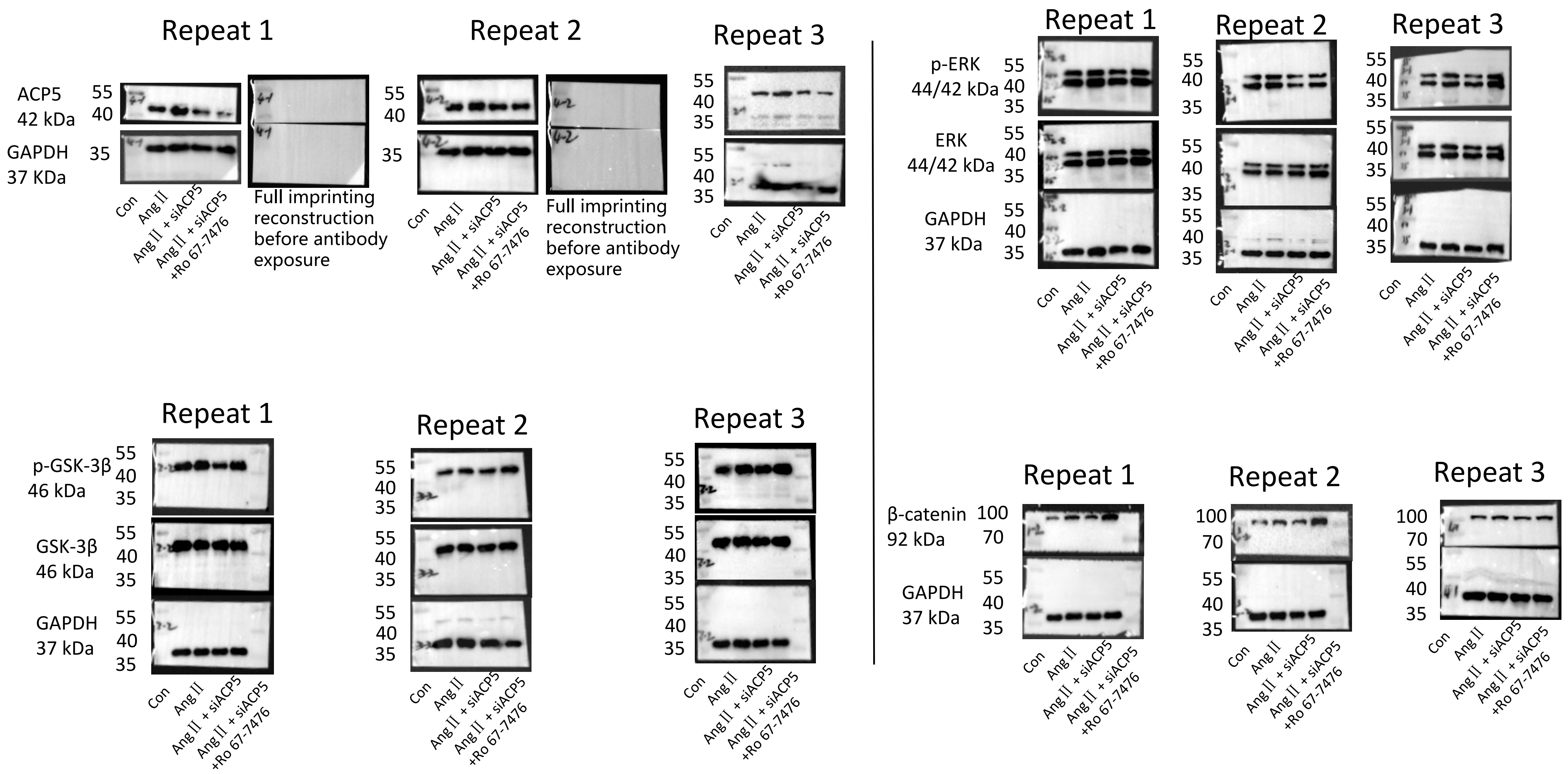

original western blot for three repeats in figure 6P

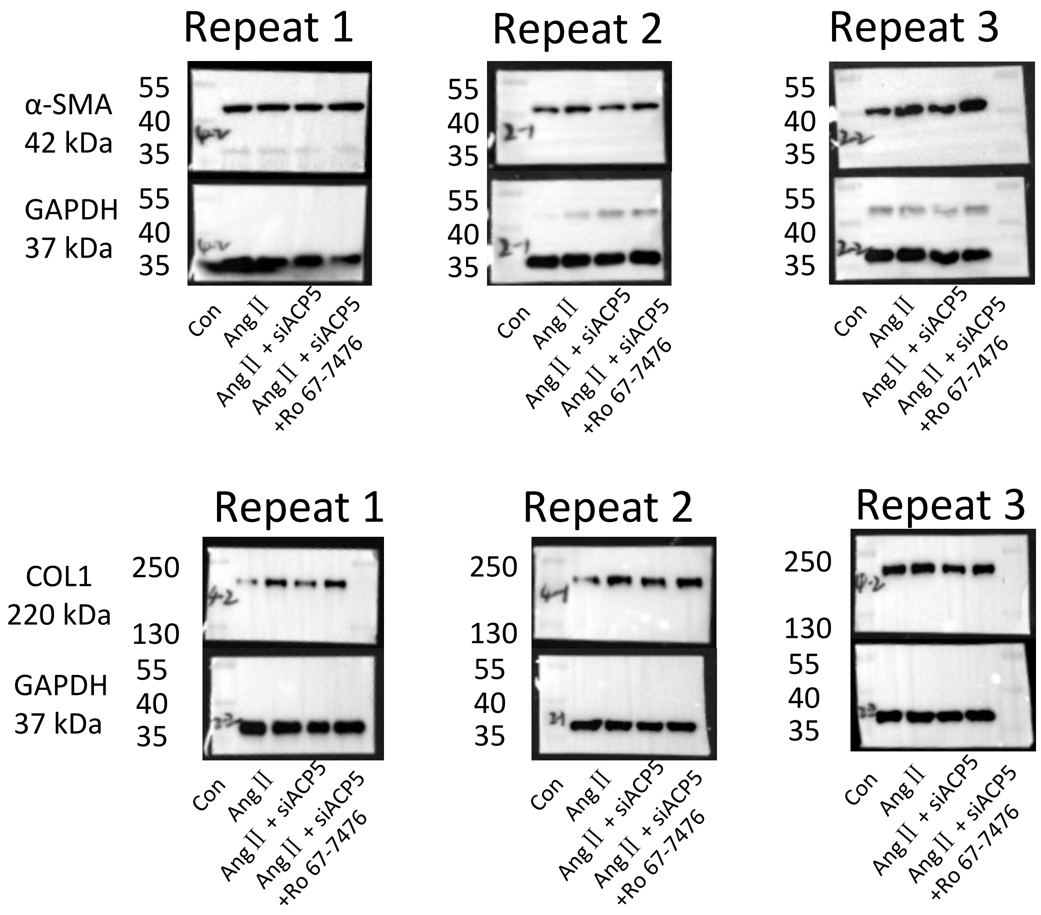

Supplement: Supplementary file 3 — Supplementary Material 3 [file 10020_2024_856_MOESM3_ESM.pdf]
