# Supplementary material for: Inhibition of tartrate-resistant acid phosphatase 5 can prevent cardiac fibrosis after myocardial infarction
Source: Mol Med. 2024 Jun 15;30:89. doi: 10.1186/s10020-024-00856-1 (PMC11179352; doi:10.1186/s10020-024-00856-1)

**Supplementary Information**

Supplementary Fig. 1


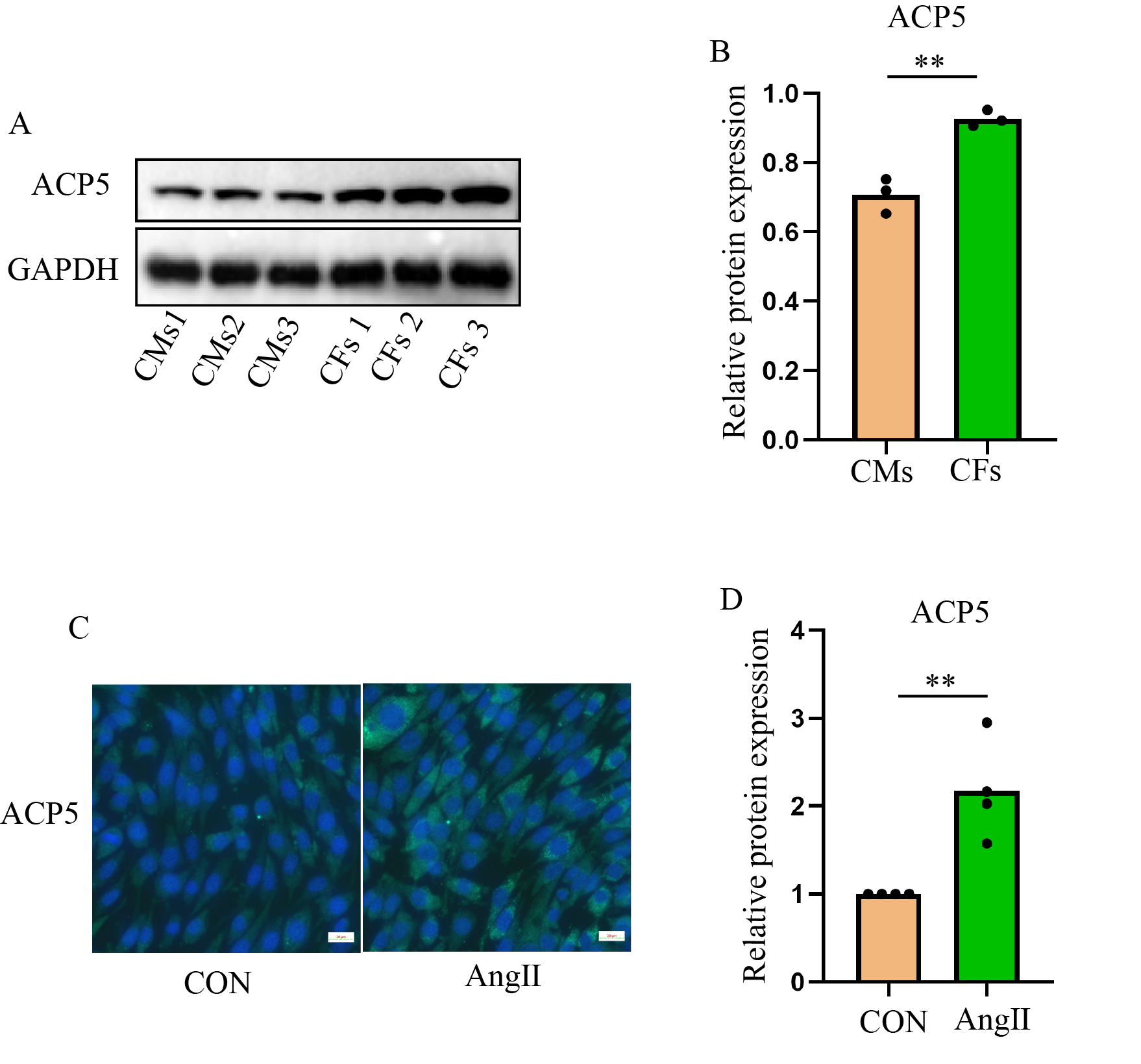


Supplementary table 1. Clinical information baseline characteristics of clinical subjects.


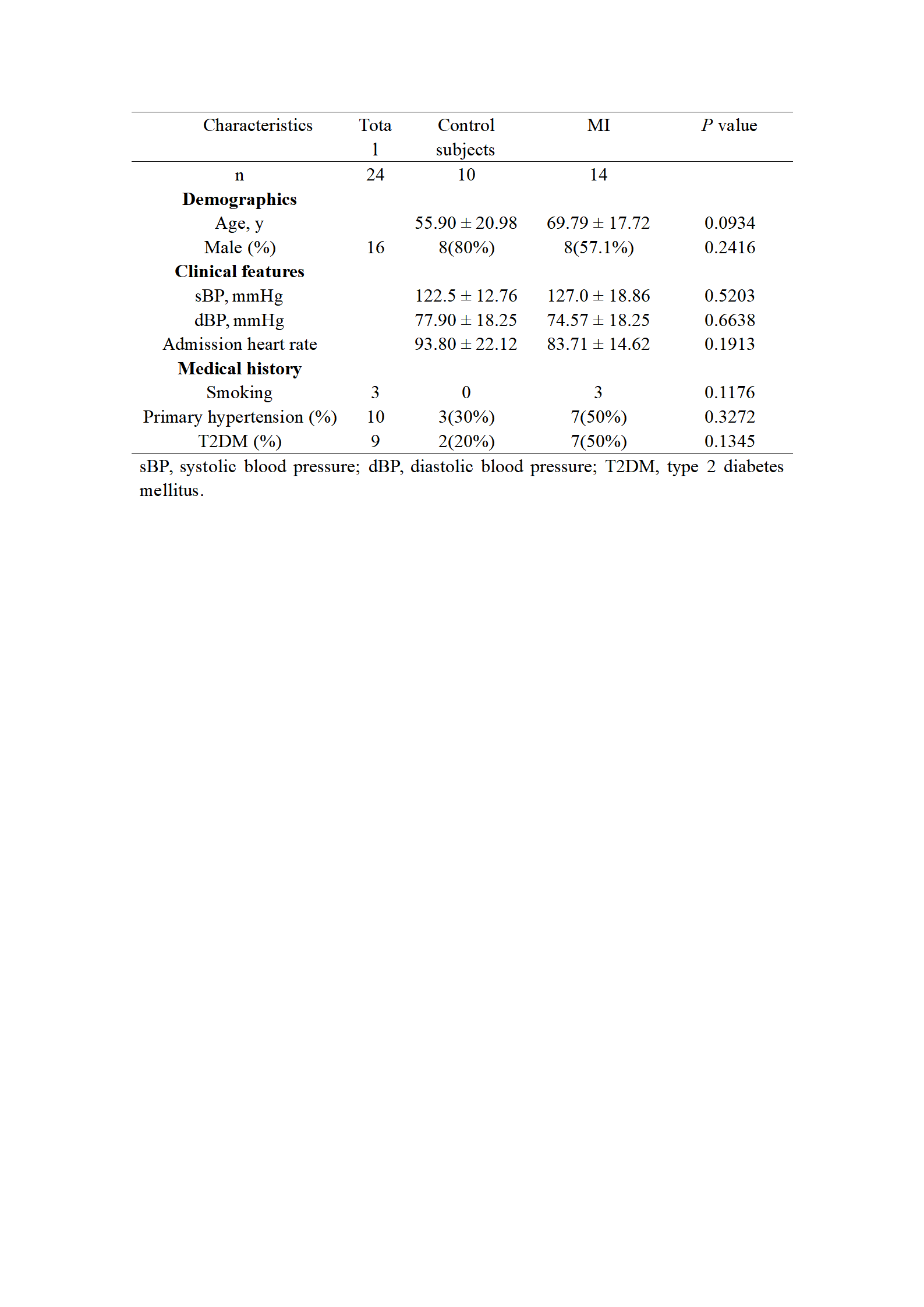

Supplement: Supplementary file 4 — Supplementary Material 4: Fig. 1 Western blot analysis of the ACP5 expression in cardiomyocytes and CFs (A-B). Immunofluorescence expression and quantitative analysis of ACP5 in CFs (C-D). **P < 0.01. table 1 Clinical information baseline characteristics of clinical subjects. [file 10020_2024_856_MOESM4_ESM.docx]
